# Next-generation risk assessment read-across case study: application of a 10-step framework to derive a safe concentration of daidzein in a body lotion

**DOI:** 10.3389/fphar.2024.1421601

**Published:** 2024-06-19

**Authors:** Abdulkarim Najjar, Jochen Kühnl, Daniela Lange, Camille Géniès, Carine Jacques, Eric Fabian, Anne Zifle, Nicola J. Hewitt, Andreas Schepky

**Affiliations:** ^1^ Beiersdorf AG, Hamburg, Germany; ^2^ Pierre Fabre Dermo-Cosmétique and Personal CareToulouse, Toulouse, France; ^3^ BASF SE, Ludwigshafen am Rhein, Germany; ^4^ Kao Germany GmbH, Darmstadt, Germany; ^5^ Cosmetics Europe, Auderghem, Belgium

**Keywords:** daidzein, genistein, PBPK, safety assessment, read-across

## Abstract

**Introduction:** We performed an exposure-based Next Generation Risk Assessment case read-across study using New Approach Methodologies (NAMs) to determine the highest safe concentration of daidzein in a body lotion, based on its similarities with its structural analogue, genistein. Two assumptions were: (1) daidzein is a new chemical and its dietary intake omitted; (2) only *in vitro* data were used for daidzein, while *in vitro* and legacy *in vivo* data for genistein were considered.

**Methods:** The 10-step tiered approach evaluating systemic toxicity included toxicokinetics NAMs: PBPK models and *in vitro* biokinetics measurements in cells used for toxicogenomics and toxicodynamic NAMs: pharmacology profiling (i.e., interaction with molecular targets), toxicogenomics and EATS assays (endocrine disruption endpoints). Whole body rat and human PBPK models were used to convert external doses of genistein to plasma concentrations and *in vitro* Points of Departure (PoD) to external doses. The PBPK human dermal module was refined using *in vitro* human skin metabolism and penetration data.

**Results:** The most relevant endpoint for daidzein was from the ERα assay (Lowest Observed Effective Concentration was 100 ± 0.0 nM), which was converted to an *in vitro* PoD of 33 nM. After application of a safety factor of 3.3 for intra-individual variability, the safe concentration of daidzein was estimated to be 10 nM. This was extrapolated to an external dose of 0.5 μg/cm2 for a body lotion and face cream, equating to a concentration of 0.1%.

**Discussion:** When *in vitro* PoD of 33 nM for daidzein was converted to an external oral dose in rats, the value correlated with the *in vivo* NOAEL. This increased confidence that the rat oral PBPK model provided accurate estimates of internal and external exposure and that the *in vitro* PoD was relevant in the safety assessment of both chemicals. When plasma concentrations estimated from applications of 0.1% and 0.02% daidzein were used to calculate bioactivity exposure ratios, values were >1, indicating a good margin between exposure and concentrations causing adverse effects. In conclusion, this case study highlights the use of NAMs in a 10-step tiered workflow to conclude that the highest safe concentration of daidzein in a body lotion is 0.1%.

## 1 Introduction

The full testing ban of the use of animals for evaluating the safety of cosmetics ingredients came into force in March 2013 (EU, 2009). Since then, an increasing number of countries outside the EU now prohibit the use of animals for testing cosmetics ingredients. Consequently, continued global efforts strive to modernize safety assessments for cosmetic ingredients and chemicals under REACH legislation without animal testing in a strategic manner that is protective of human health. Despite the common aim to implement non-animal assays for chemical registration, some cosmetics ingredients registered under REACH regulations have been tested in animal assays to comply with the requirements by ECHA for toxicity data and worker safety assessments (Fentem et al., 2021; Knight et al., 2021). Rather alarmingly, the alternative methods submitted in some dossiers were deemed insufficient by ECHA, who requested *in vivo* studies instead. This highlights the need to gain the confidence of different regulatory bodies to show that Next-Generation Risk Assessments (NGRAs) using so-called “New Approach Methodologies” (NAMs) are at least equally effective and protective as traditional risk assessments using animal studies.

While several validated NAMs exist to replace assays evaluating local effects of cosmetics e.g., skin sensitization, systemic effects are more difficult to predict due to the multitude of mechanisms and target organs involved. Additionally, prediction of systemic effects requires consideration of biokinetics of the parent and/or metabolites once they enter the circulation. Since systemic toxicity is difficult to predict, the strategy for evaluating the safety of cosmetic ingredients has changed to use NAMs in an exposure-driven approach to derive safe concentrations that are protective of human health (rather than predictive) (Hatherell et al., 2020). In line with this paradigm shift, the Cosmetics Europe Long Range Science Strategy (LRSS) has performed several case studies to evaluate the practical application of NGRAs for cosmetic ingredients (Desprez et al., 2018; OECD, 2021; Alexander-White et al., 2022; Ouedraogo et al., 2022). These case studies included read-across assessments of systemic toxicity for chemicals with an available suitable analogue and *ab initio* assessments for chemicals without analogues for which the NGRA must be conducted using NAM data only. The present work is a read-across case study to determine the highest concentration at which daidzein can be added as an ingredient to a body lotion, based on its similarities with the data-rich toxicological profile of a structural analogue, genistein, present in soya extract. The aim was to establish a proof-of-concept for the value added by NAMs in read-across, whereby *in silico* information, *in vitro* toxicodynamic and toxicokinetic data are used to support the biological similarity of analogues and establish potency. Only the systemic toxicity of daidzein was investigated here, whereas other local endpoints such as eye irritation, skin sensitization/irritation and phototoxicity, were not considered since there are established alternative NAMs with OECD Test Guidelines to address these. Two main assumptions were made: (1) daidzein was assumed to be a new cosmetic ingredient and knowledge of its presence in soya extract ignored; (2) only *in vitro* data available in the literature were used for daidzein, while all *in vitro* and legacy *in vivo* data for its analogue, genistein, were considered. The case study was guided by the SEURAT-1 safety assessment workflow described by Alexander-White et al. (2022) and Berggren et al. (2017) and according to the International Cooperation on Cosmetics Regulation (ICCR) NGRA principles (Dent et al., 2018), with the aim to use only non-animal approaches to assure the systemic safety of this ingredient. NAMs employed included: Physiologically-based pharmacokinetic (PBPK) modelling, cell stress assays, pharmacology profiling (i.e., the ability to interact with specific molecular targets), transcriptomics and “estrogen-, androgen-, thyroid signaling and steroidogenesis” (EATS) assays (endpoints for endocrine disruption). An analysis of sources of uncertainty in the safety assessment was also conducted.

Genistein is a naturally occurring compound found exclusively in soybeans and other legumes and structurally belongs to a class of compounds known as isoflavones. It inhibits protein-tyrosine kinase and topoisomerase-II activity and is used as an antineoplastic and antitumor agent. Recent research has shown the potential for the use of genistein in medicine for menopausal relief, osteoporosis, blood cholesterol, and lowering the risk of some hormone-related cancers (Mukund et al., 2017; Maliehe et al., 2019; Thangavel et al., 2019). Genistein has potential endocrine disruption properties used in cosmetics (listed by the EU Commission 2019) and since daidzein has a similar structure, it was evaluated for its potential to also cause endocrine disruption. This NAM-based concept is also applicable to other toxicological effects; therefore, the aim of this case study is to show that NAMs are (a) relevant and sufficient for high quality safety assessment, (b) provide valuable support to defend these compounds and (c) to eventually foster their regulatory acceptance e.g., by the Scientific Committee for Consumer Safety (SCCS). Higher tier approaches are needed to refine risk assessment.

## 2 Materials and methods

**FIGURE 1:**
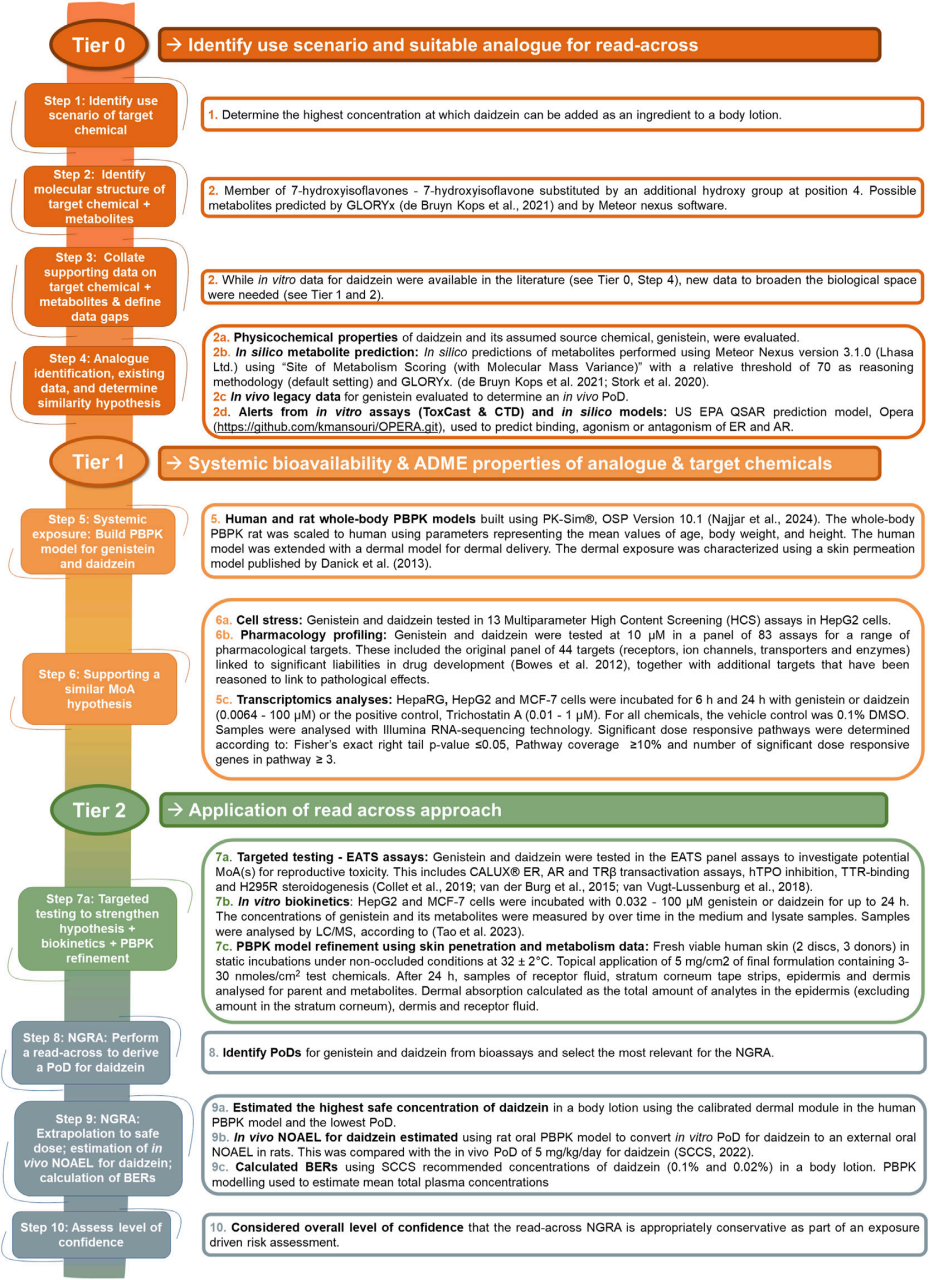
Overview of the *in silico* and *in vitro* methods used in the tiered workflow for the read-across NGRA for daidzein.

The safety assessment was conducted in a tiered fashion according to the workflow described by Desprez et al. (2018). An overview of the *in silico* and *in vitro* methods used in the tiered workflow is shown in Figure 1 and details of the protocols are described in Supplementary Materials S1. The PBPK models for genistein and daidzein are described in detail by Najjar et al. (2024).

## 3 Results and discussion

### 3.1 Tier 0: identify use scenario and suitable analogue for read-across

#### 3.1.1 Tier 0, step 1: identify use scenario for target chemical, daidzein

Rather than using NAMs to support the use of a predetermined exposure scenario, this case study aimed to determine the highest concentration at which daidzein can be added as an ingredient to a body lotion. This meant that exposure-based waiving of further tests using the Threshold of Toxicological Concern (TTC) concept was not applicable in this case study. The amount of a leave-on body lotion applied daily is 7.82 g/day, equivalent to 123.2 mg/kg bw/day considering a typical human body weight of 60 kg (SCCS, 2023).

#### 3.1.2 Tier 0, step 2: identify molecular structure of target chemical and its metabolites

**FIGURE 2:**
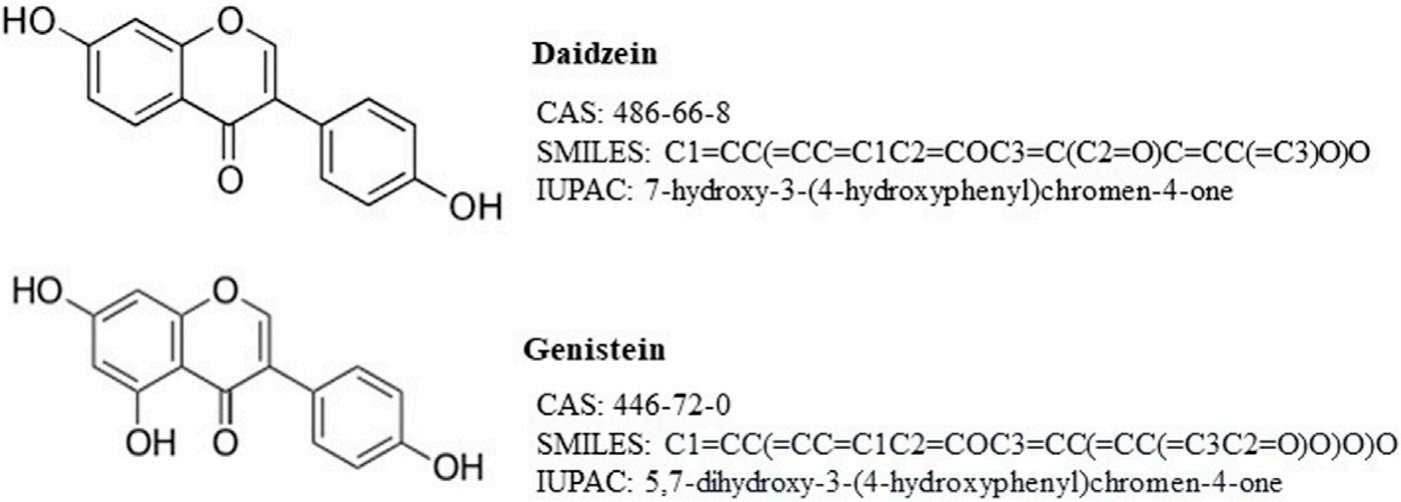
Structures and identifiers of daidzein and genistein.

The structure and identifiers of the target chemical, daidzein, are shown in Figure 2. It is a member of the class of 7-hydroxyisoflavones, whereby 7-hydroxyisoflavone is substituted by an additional hydroxy group at position 4'.

Open-source (GLORYx (de Bruyn Kops et al., 2021)) and commercial (Meteor nexus) prediction software were used to predict the likely metabolites of daidzein (Supplementary Materials S2). Both software models predicted the formation of glucuronide and sulfate conjugates with a high probability. These were also identified and confirmed to be formed in several *in vitro* hepatic metabolism assays (Pritchett et al., 2008; Toro-Funes et al., 2015; Tao T.-P. et al., 2023; Tao T. P. et al., 2023). GLORYx predicted a low probability for the formation of a methyl metabolite and neither software models predicted the formation of a hydroxylated metabolite. None of these metabolites were detected in *in vitro* incubations (Pritchett et al., 2008; Toro-Funes et al., 2015; Tao T.-P. et al., 2023; Tao T. P. et al., 2023).

#### 3.1.3 Tier 0, step 3: collate supporting data on target chemical and its metabolites, and define data gaps

As mentioned, there were two main assumptions in the case study: (1) daidzein was assumed to be a new cosmetic ingredient and knowledge of its presence in soya extract ignored; (2) only *in vitro* data available in the literature were used for daidzein, while all *in vitro* and legacy *in vivo* data for its analogue, genistein, were considered. This meant that it was assumed that there were no *in vivo* pharmacokinetics or toxicodynamics data for daidzein. While *in vitro* data for daidzein were available in the literature (see Tier 0, Step 4), new data to broaden the biological space were needed (see Tier 1 and 2).

#### 3.1.4 Tier 0, step 4: analogue identification, existing data and determine similarity hypothesis

##### 3.1.4.1 Identification of genistein as a suitable analogue

The starting hypothesis for the read-across was based on the high similarity in the chemical structures, and that the target chemical, daidzein, will have a similar bioavailability and bioactivity to the source chemical, genistein. The structures of daidzein and genistein are very similar, with the only difference being an additional hydroxyl group on genistein compared to daidzein (Figure 2). The physicochemical properties of chemicals affect their bioavailability and, consequently, biological responses observed *in vitro* or *in vivo*. Therefore, the similarities of the physicochemical properties of daidzein and its assumed source chemical, genistein, were evaluated (Table 1). These indicate that the chemicals are similar and, importantly, that a PBPK model for genistein could be used to build a model for daidzein (see Section 3.2 on PBPK modelling).

**TABLE 1:** Physicochemical Properties of genistein and daidzein relevant to bioavailability.

Property	Genistein	Daidzein	Unit/format	Protocol	Reference
Log P_ow_	3.04	3.3		*In silico*	Drugbank (https://go.drugbank.com)
Boiling point	555.5	512	°C	*In vitro*	ChemSpider
Melting point	301.5	323	°C	*In vitro*	PubChem
Vapor pressure	1.33 × 10^−9^	3.46 × 10^−10^	mmHg	*In silico*	OPERA (Mansouri et al., 2018)
Molecular weight	270.24	254.23	g/mol		PubChem
Water solubility	0.12	0.053	mg/mL	*In silico*	ADMET Predictor 10 (SimulationsPlus, 2024)
pKa (dissociation constant)	7.25 ± 0.84	7.51 ± 0.07	at 25°C	*In vitro*	Nan et al. (2014)
		9.47 ± 0.14			
Relative Density	1.5 ± 0.1	1.4 ± 0.1	g/cm^3^	*In silico*	ChemSpider, (ACD/labs)

Open-source [GLORYx (de Bruyn Kops et al., 2021)] and commercial (Meteor nexus) prediction software were used to predict the likely metabolites of genistein (Supplementary Materials S2). As for daidzein, the software models predicted a high probability of the formation of two glucuronides and a sulfate metabolite, which were confirmed to be formed in several *in vitro* hepatic MetID assays (Bursztyka et al., 2008). Likewise, GLORYx predicted a low probability for the formation of a methyl metabolite and Meteor nexus predicted the possibility of a hydroxylated metabolite; however, neither of these were identified in *in vitro* hepatic metabolism assays. These predictions were confirmed by reports on clinical studies of genistein in humans (Yang et al., 2012) and *in vitro* metabolism data (Bursztyka et al., 2008; Pritchett et al., 2008).

The use of genistein as a source chemical for daidzein was supported by comparing the quality of different potential analogues using ToxGPS (Version 4) software (Yang et al., 2023). The analogue quality considers chemical similarities using MACCS and ToxPrint Fingerprints, Chemotype profiles, molecular properties, including quantum mechanical parameters, and Skyline profiles. This evaluation indicated that the closest analogue to daidzein was genistein, with an analogue quality of 0.95 (See Supplementary Materials S3).

##### 3.1.4.2 *In vivo* legacy data for genistein and rationale for selection of a NOEL for the PoD

*In vitro* and *in vivo* mutagenicity and genotoxicity data were available for genistein (see SCCS (2022) and summary of the results in Supplementary Materials S4). In *in vitro* assays, this chemical was positive for gene mutations in mammalian cells but negative in bacterial cells. In addition, genistein was negative for gene mutations in studies with Big Blue transgenic rats, indicating that it does not cause gene mutations *in vivo*. While the results of *in vitro* assays for aneugenicity and clastogenicity for genistein were inconclusive, *in vivo* micronucleus and chromosomal aberration studies with genistein showed no clastogenicity.

The no observed adverse effect level (NOAEL) values for genistein from repeat dose studies were above 50 mg/kg/day. Increases in organ weights were observed at the high dose of 500 mg/kg/day in male rats (kidney, spleen, adrenal and testes) and females (liver, kidney, spleen, ovary and uterus) (McClain et al., 2006). NOAELs in reproductive studies were much lower (see Supplementary Materials S5). In the reproductive dose study in which genistein was administered in the feed to Sprague-Dawley rats (NTP, 2007), there were effects on prostate and pituitary gland weights at higher doses of 1,250 ppm, and ductal/alveolar hyperplasia and hypertrophy of the mammary glands of males at ≥25 ppm. No effects were observed at 5 ppm. In the multi-generation study, male rats exposed to 100 or 500 ppm were also observed to have increased rates of mammary gland hyperplasia. Therefore, the Point of Departure (PoD) was based on the lowest NOAEL of 0.3 mg/kg (5 ppm) in male rats. The LOAEL was 7 mg/kg for male rats, which was also noted by the SCCS in the latest opinion on genistein (SCCS, 2022).

##### 3.1.4.3 *In silico* alerts for genistein and daidzein

In addition to the evaluation of physicochemical properties and available *in vitro* data, an analysis of *in silico* data that are deemed relevant to the endpoint for the read-across are important in confirming the similarity and suitability of identified analogs. Based on the conclusions from *in vitro* and legacy *in vivo* mutagenicity and genotoxicity studies on genistein, daidzein was considered to have no genotoxicity potential. Therefore, in this case study, the *in silico* alerts related to the reported endocrine properties observed in the *in vivo* studies for genistein were evaluated. Therefore, profilers that the OECD Quantitative structure–activity relationship (QSAR) Toolbox highlights as relevant for reproductive toxicity i.e., the Developmental And Reproductive Toxicology (DART) scheme, estrogen receptor (ER) binding, Retinoic Acid Receptor binding and the rtER Expert System from the United States Environmental Protection Agency (US EPA), were evaluated to examine the similarity between genistein and daidzein. Results showed that the two compounds were similar with respect to DART and ER binding properties (Table 2). Both chemicals also share the same predictions for “Retinoic Acid Receptor Binding” and “rtER Expert System US EPA,” with negative results or both being out-of-domain, respectively.

**TABLE 2:** *In silico* profilers relevant to reproductive toxicity. Profiling results obtained from OECD QSAR Toolbox v 4.2.

Chemical	DART scheme	ER binding	Retinoic acid receptor binding	rtER expert system US EPA
Genistein	Known precedent reproductive and developmental toxic potential	Very strong binder, OH group	Not possible to classify according to these rules	No alert found
	Non-steroid nucleus derived ER and AR			
	Non-steroid nucleus derived ER and AR >> Flavone and mycoestrogen related derivatives (2b-1)			
Daidzein	Known precedent reproductive and developmental toxic potential	Very strong binder, OH group	Not possible to classify according to these rules	No alert found
	Non-steroid nucleus derived ER and AR			
	Non-steroid nucleus derived ER and AR >> Flavone and mycoestrogen related derivatives (2b-1)			

Profilers and (applicability to endpoint): DART, scheme (Developmental and Reproductive Toxicity), ER, binding (Toxicity to Reproduction), Retinoic Acid Receptor Binding (Toxicity to Reproduction) rtER, expert system (Toxicity to Reproduction).

The US EPA QSAR prediction model, Opera, was used to predict the binding, agonism or antagonism of ER and androgen receptor (AR) by genistein and daidzein (both are in the applicability domain of the model). The results are reported as binary (either active or inactive), rather than a grading of binding or activity (shown in Supplementary Materials S6). Genistein and daidzein were predicted to bind to the ER and to be active as an agonist and antagonist for this receptor. Both chemicals were also predicted to bind to the AR but were active as an antagonist for this receptor (i.e., inactive for agonism).

After oral administration, genistein enters the systemic circulation mainly as a glucuronide conjugate (Yang et al., 2012); therefore, it was considered whether the metabolites of genistein and daidzein should be tested in *in vitro* assays. While glucuronidation and sulfation are generally thought to be detoxifying pathways, the docking of the metabolites to the estrogen, androgen, thyroid and other nuclear receptors was evaluated using the open-source endocrine Disruptome tool (http://endocrinedisruptome.ki.si/). Genistein and daidzein were predicted by the molecular docking tool to bind to the AR, ERα and the Mineralocorticoid Receptor (Supplementary Materials S7). By contrast, their metabolites were predicted to have a low probability of binding to the same receptors. One glucuronide of genistein (but not daidzein) was indicated to bind to the glucocorticoid receptor, which would require follow-up investigations. None of the predicted metabolites were predicted to bind with a high probability to any of the receptors evaluated in the molecular docking program. The conclusion from this analysis was that the metabolites do not need to be tested in the safety screen or toxicogenomics assays. This is a good example of the use of a NAM to follow up on the impact of metabolism on toxicity.

##### 3.1.4.4 *In vitro* alerts for genistein and daidzein

**FIGURE 3:**
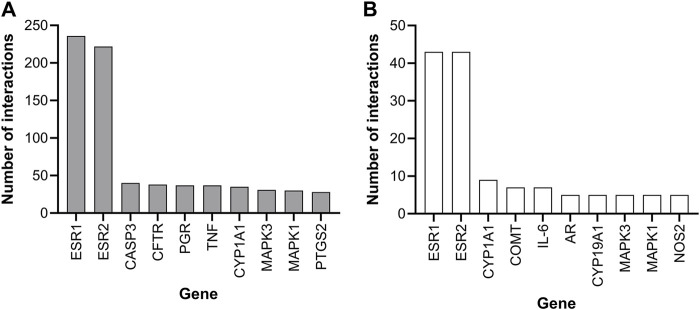
CTD database ten genes with the most curated interactions for **(A)** genistein and **(B)** daidzein.

The Comparative Toxicogenomics Database (CTD, https://ctdbase.org) indicated that genistein and daidzein both affect the ER pathway, with the most curated genes being reported for the ER genes, ESR1 (coding for ERα) and ESR2 (coding for ERβ) (Figure 3). Other gene-protein interactions shared by both chemicals were MAPK1/3 and CYP1A1, which were considered not sufficiently specific to identify a Mode of Action (MoA) since they are involved in many different pathways. CTD data does not comprise the full genome for daidzein; therefore, a full transcriptomics analysis was required, along with the pharmacology profiling screen to determine whether the main effect of both chemicals is on the ER pathway or whether other organs could be targets (see Section 3.1.4.4 Transcriptional profiling).

ToxCast data were available for genistein and daidzein tested in EATS assays (See Supplementary Materials S8). These data indicated that the two main pathways affected are the estrogen and thyroid pathways and that the biological activity of daidzein is at least an order of magnitude lower than genistein.

##### 3.1.4.5 Summary of tier 0 and next steps

Read-across based on high similarity in chemical structures, metabolites and physicochemical properties to substantiate the suitability of genistein as the source comparator for daidzein. Predicted formation of glucuronide and sulfate by *in silico* models were identified by several *in vitro* MetID assays. *In silico* and *in vitro* toxicodynamic data indicate that the two main pathways affected are the estrogen and thyroid pathways and that the biological activity of daidzein is at least an order of magnitude lower than genistein based on the outcome of the ToxCast EATS assay panel. The profilers highlighted as relevant for reproductive toxicity i.e., the DART scheme, ER binding, Retinoic Acid Receptor binding and the rtER Expert System from US EPA [in the OECD QSAR Toolbox v3.4 (Webster et al., 2019)], showed that genistein and daidzein were similar with respect to DART and ER binding properties. None of the metabolites were predicted by the molecular docking tool to bind to receptors relevant to ED–only the parent chemicals, genistein and daidzein, were predicted to have a high probability of binding to AR, ER and MR. *In vivo* legacy data for genistein were evaluated, from which a multi-generation study was selected for the PoD. The lowest NOAEL of 0.3 mg/kg (5 ppm) was derived for male rats. Based on *in vitro* and *in vivo* studies, it was concluded that genistein does not cause mutagenicity or genotoxicity *in vivo*. Therefore, follow up *in vitro* assays to investigate the mutagenicity or genotoxicity of daidzein were not conducted.

### 3.2 Tier 1: systemic bioavailability and ADME properties of analogue and target chemicals

#### 3.2.1 Tier 1, step 5: systemic bioavailability and ADME parameters of analogue and target chemicals

Understanding absorption, distribution, metabolism, and excretion (ADME) properties and the relative rate and extent of biotransformation of genistein and daidzein is important in the examination of potential potency differences across the category members that could arise from differences in bioavailability and internal exposure levels. The main *in vitro* ADME properties of the two chemicals relating to systemic metabolism and clearance after oral application are similar, which meant that the PBPK model for genistein could be used as a basis for the daidzein model (Najjar et al., 2024).

To provide an estimation of the *in vitro* PoD for the bioactivity assays, a PBPK model was built to convert the external NOAEL dose of genistein to an internal plasma concentration (C_max_, described by Najjar et al. (2024). As explained in Section 3.1.4.2, the PoD for genistein was based on the NOAEL of 0.3 mg/kg/day in a reproductive toxicity in the rat following oral administration. The rat PBPK model for genistein was then used to estimate the steady state plasma concentrations over 7 days at the NOAEL dose of 0.3 mg/kg/day. The resulting mean C_max, total_ was estimated at 24.1 nM, whereas the C_max, total_ (CI5-95%) ranged from 12.4 to 61.5 nM. The mean C_max,fu_ was estimated at 0.48 nM, where the C_max,fu_ (CI5-95%) ranged from 0.38 to 1.37 nM.

The genistein rat model was validated since it was able to reproduce the observed C_max_ values of genistein an *in vivo* pharmacokinetics study (Najjar et al., 2024). This indicates that the PBPK model can be used to estimate the internal dose metrics (C_max_) of genistein associated with the NOAEL. Thus, the model was further extrapolated to build a rat PBPK model for daidzein. The daidzein model was then used to simulate plasma concentrations after repeated doses of 0.3 mg/kg/day. The estimated mean C_max, total_ of 45.17 nM (CI 5%–95% = 17.14–135.9 nM) of daidzein was higher than the estimated C_max_ for genistein (the mean C_max,fu_ was estimated at 1.33 nM (CI 5%–95% = 0.72–2.61 nM)). These values were used to set the doses for the toxicogenomics assays (Doses: 100, 20, 4, 0.8, 0.16, 0.032, and 0.0064 µM) and the cell stress assays (300, 75, 18.75, 4.69, 1.17, 0.29, 0.073, and 0.018 µM).

#### 3.2.2 Tier 1, step 6: supporting a similar MoA hypothesis.

##### 3.2.2.1 Cell stress assays

Genistein and daidzein were tested in a panel of cell stress assays, which were analyzed using high content screening. None of the concentrations of genistein and daidzein tested (0.0061–100 µM) caused overt toxicity, assessed according to lactate dehydrogenase leakage. The targets have been shown to be predictive for compounds causing various forms of toxicity (Baltazar et al., 2020; Hatherell et al., 2020). The protocol and results from these assays are presented in Supplementary Materials S1.5, S9, respectively). Neither chemical caused marked responses in any of the assays. The lowest minimum effective concentration (MEC) of genistein was 11.6 µM, which resulted in a decreased mitochondrial membrane potential. This endpoint was also the most sensitive for daidzein, for which the MEC was 10.8 µM. This effect could indicate mitochondrial toxicity but may also be an adaptive response to cellular energy demands. The conclusion from this assay was that genistein and daidzein were not causing cell stress at concentrations below 10 µM.

##### 3.2.2.2 Pharmacology profiling

Cosmetics Europe’s Systemic Toxicity Taskforce (SysTox-TF) has extended the list of targets in an *in vitro* screen originally described by Bowes et al. (2012) to assess the effects of cosmetic chemicals on target molecules. The approach was based on the knowledge that various targets of pharmacological interest have been linked to human adverse drug reactions, and that screening of these has helped the pharma industry in identifying drug candidates with acceptable human safety profiles (Bowes et al., 2012; Scott et al., 2022). The methods used by the pharma industry were adapted for use in cosmetic chemical NGRA. A final list of 83 target assays were considered suitable for cosmetic chemical safety testing (see Supplementary Materials S1.7). The targets were receptors, ion channels, transporters and enzymes identified by the pharma industry as linked to human adverse drug reactions, supplemented by additional targets that have been reasoned to be linked to systemic toxicities of chemicals in animals. Chemicals that are flagged for a target are tested in follow-up dose-response assays to establish potency of the interaction. Out of the 83 assays, there were 11 hits for genistein and 3 hits for daidzein (Table 3). Of note, one of the targets was in common with Chip2 toxicogenomics data for genistein (Tao T.-P. et al., 2023), namely, carboxylesterase-2 (COX2). 5-HT2A and 5-HT2B were found to be promiscuous targets, such that it was a common target to which many chemicals bound (Bowes et al., 2012; Valentin et al., 2018; Dodson et al., 2021). Of the targets in common to both chemicals, daidzein was less potent than genistein and as expected, the ER hormone receptors were identified as the most potently affected hits.

**TABLE 3:** Comparison of the target hits and potency of genistein and daidzein in the pharmacology screen. The targets which were hits are denoted by underlined values. (h) = human, RL = radioligand assay. A full concentration response curved was not applicable for targets which were not hits.

Assay NAME	Genistein		Daidzein	
	% inhibition @10 µM	IC_50_ (µM)	% inhibition @10 µM	IC_50_ (µM)
Estrogen ERα (h) (agonist RL)	101.07	0.14	100.24	0.308
ERβ Human Estrogen NHR Binding (Agonist)	104.33	0.00308	100.00	0.02472
EGFR Human RTK Kinase Enzyme activity	71.05	5.984	17.78	Not applicable
MAO-A (antagonist RL)	85.95	2.12	49.64	9.18
5-HT2A (h) (agonist RL)	57.31	7.60	38.85	Not applicable
5-HT2B (h) (agonist RL)	91.89	0.682	44.11	Not applicable
Adenosine A1 (h) (antagonist RL)	53.51	9.12	19.92	Not applicable
Adenosine A2A (h) (agonist RL)	52.21	10.0	20.50	Not applicable
COX1 (h)	70.76	7.69	44.74	Not applicable
COX2 (h)	53.81	13.6	21.63	Not applicable
PDE4D2 (h) (phosphodiesterase 4D)	87.03	0.738	0.04	Not applicable

**FIGURE 4:**
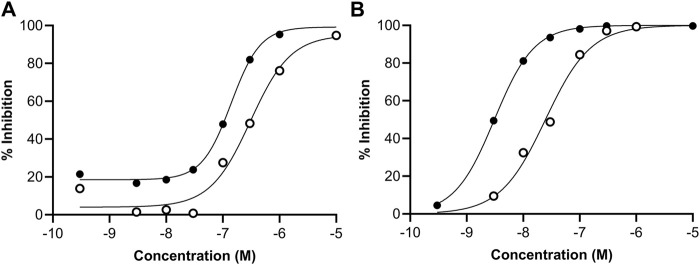
Concentration-response curves for the agonism of genistein (black circles) and daidzein (white circles) on the **(A)** ERα and **(B)** ERβ receptors measured in the pharmacology profiling assays.

Follow-up dose response curves were conducted for all targets that were inhibited by 50% or more and the IC_50_ values determined (Table 3). The dose response curves for ERα and ERβ are shown in Figure 4. The difference in the potency of genistein and daidzein observed in the EATS assays from ToxCast were also evident in these assays. While the IC_50_ and Ki values for ERα agonism were lower for genistein, the Lowest Observed Effect Concentrations (LOECs) were similar (44 nM for genistein and 35 nM for daidzein) (Table 4). The LOEC is defined as the lowest concentration where the compound significantly activates the assay, which is set to 10% of the maximum reference compound activity for agonist assays. The most potent effect was observed for the agonist binding to ERβ, with IC_50_ values for genistein and daidzein of 3.1 nM and 24.7 nM, respectively, and Ki values for genistein and daidzein of 0.64 nM and 5.1 nM, respectively. The LOECs for genistein and daidzein on ERβ were similar (0.518 nM and 3.25 nM, respectively).

**TABLE 4:** Summary of the potency of binding of genistein and daidzein to ERα and ERβ. Values were derived from the concentration-response curves shown in Figure 4.

	Genistein		Daidzein		Reference control (diethylstilbestrol)	
	ERα	ERβ	ERα	ERβ	ERα	ERβ
IC_50_ (nM)	140	3.1	308	24.7	NA	1.18
Ki (nM)	40	0.64	87.9	5.1	NA	0.24
LOEC (nM)	44	0.52	35.2	3.2	NA	NA

The Ki value is a relevant parameter with which to compare the potency between the effect on ERα and ERβ, since this takes into account the relative amount of ligand in the assay (which may be different). The Ki values for genistein in the ERα and ERβ assays were 40 nM and 0.64 nM, respectively, and the Ki values for daidzein in the ERα and ERβ assays were 87.9 nM and 5.1 nM, respectively. Notably, the Ki for genistein in the ERβ assay was similar to that of the reference control, diethylstilbestrol, indicating the high potency of this chemical.

##### 3.2.2.3 Transcriptomics analyses

**FIGURE 5:**
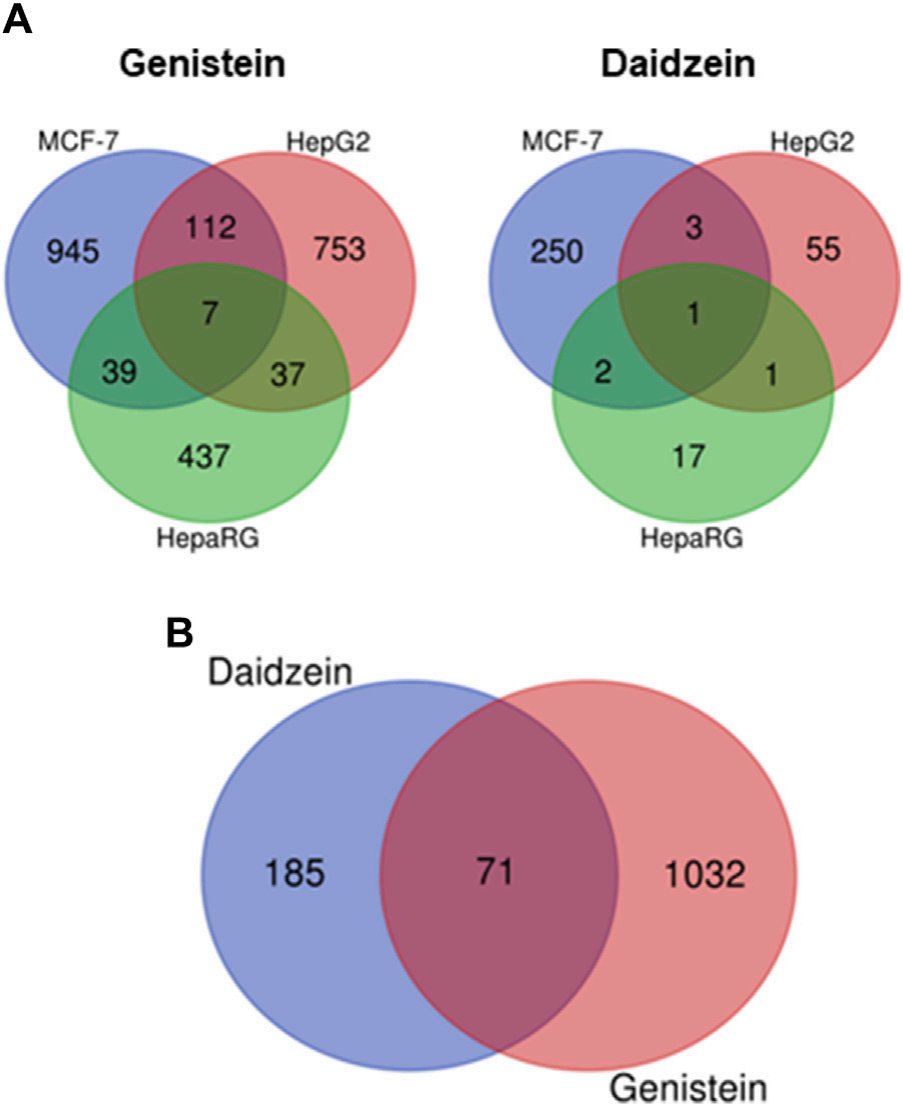
Comparison of effect of genistein and daidzein on the gene expression profiles of different cells. **(A)** Different cell types: deregulated genes in MCF-7, HepG2 and HepaRG cells treated with genistein and daidzein, **(B)** Deregulated genes in MCF-7 treated with genistein and daidzein.

A transcriptomics approach was used to complement the pharmacology profiling data and inform on the relative potency of genistein and daidzein (see Supplementary Materials S1.8). The cell types tested i.e., MCF-7, HepG2 and HepaRG, are considered to provide a broad biological coverage, with HepaRG cells enabling an evaluation of the impact of metabolism on gene changes (Baltazar et al., 2020). MCF-7 cells express functional ER and have been used to investigate ER associated transcriptional changes (Falany and Falany, 1997; Comsa et al., 2015; Yeakley et al., 2017; Matteo et al., 2023); therefore, this cell line was important for the toxicogenomics analysis, considering the *in silico* alerts and the results from the pharmacology profiling screen indicating the key role of ER in the bioactivity of genistein and daidzein. Cells were incubated with a range of concentrations (0.0064–100 µM) of genistein and daidzein for 24 h. None of the treatments resulted in a preferential up- or downregulation of genes and pathways (data not shown). The number of significantly deregulated (up- or downregulation) genes in MCF-7, HepG2 and HepaRG cells treated by genistein and daidzein are shown in Figure 5A. The total number of significantly dose responsive genes deregulated (up- and downregulated) in each cell type was lower after treatment with daidzein compared to genistein (Figure 5A). The same was true for the total number of responsive pathways deregulated (data not shown). There were 71 genes in MCF-7 cells which were deregulated by genistein and daidzein (Figure 5B). However, further analysis is needed to cover all perturbated genes measured in the experiment to justify the biological similarity.

*In vitro* PoDs were derived based on changes in gene expression pathways (Table 5). In a separate LRSS case study, there were two slightly different methods used by two partner biostatistician companies to identify deregulated pathways. These differed with respect to the number of genes passing all filters in each pathway (3 versus 5 genes). While the use of 5 genes provides higher confidence that this MoA is biologically relevant, the use of 3 genes per pathway provides a lower and thus more conservative PoD. Therefore, we captured pathways which fulfilled both criteria. In Table 5, the median Benchmark doses (BMDs) for effects on estrogen were also captured to determine whether these values were similar to the lowest PoD but, in all cases, these were higher than the lowest median BMD. The lowest median BMD for all three cell types was always linked to genistein treatment, which was up to 6-, 2- and 828-fold lower than the median BMD for daidzein in MCF-7, HepG2 and HepaRG cells, respectively. The lowest median BMDs for daidzein and genistein were 38 nM and 6.5 nM, respectively (both in MCF-7 cells but for different pathways). Interestingly, the BMD for daidzein effects on the estrogen pathway in MCF-7 cells is ∼3-fold lower than for genistein; however, the pathways affected by the two chemicals were different and were downregulated at the time points analyzed. While daidzein had no significant effects on the estrogen pathway in HepG2 or HepaRG cells, genistein had opposing effects on estrogen-dependent gene expression in these cells.

**TABLE 5:** Median BMD levels for genes representing the average 20 gene ontology pathways. The unit is in μM. The BMD relating to estrogen effects was found by searching for “estrog” in the BMD column filter with <5 genes passing all filters. The arrows denote whether the pathway was up- (↑) or down- (↓) regulated.

Chemical	Cell type	BMD median (µM)			
		No. genes passing all filters	Lowest median BMD	Pathway	BMD relating to estrogen effects
Daidzein	MCF-7	3	0.038	Positive regulation of insulin-like growth factor receptor signaling pathway (↓)	21.3 - response to estrogen ↓
		>5	0.084	Epidermal growth factor receptor signaling pathway (↓)	
	HepG2	3	16.2	C21-steroid hormone metabolic process (↑)	None
		>5	30.0	Steroid biosynthetic process (↑)	
	HepaRG	3	33.1	Arachidonic acid monooxygenase activity (↓)	None
		>5	39.3	Oxidoreductase activity (↓)	
Genistein	MCF-7	3	0.0065	L-leucine transmembrane transporter activity (↑)	60.1 - estrogen 2-hydroxylase activity ↓
		>5	0.051	Negative regulation of Notch signaling pathway (↓)	
	HepG2	3	NA	[All deregulated pathways had >5 genes]	53.8 - Estrogen-dependent gene expression ↓
		>5	15.7	Regulation of glomerular mesangial cell proliferation (↓)	
	HepaRG	3	0.040	Regulation of intracellular sterol transport (↑)	0.11- Estrogen-dependent gene expression ↑
		>5	0.051	Translation initiation complex formation (↑)	

### 3.3 Tier 2: application of read across approach

#### 3.3.1 Tier 2, step 7A: targeted testing to strengthen hypothesis and biokinetics

##### 3.3.1.1 Targeted testing of endocrine activity using EATS assays

The *in silico* and *in vitro* alerts described in Sections 3.1.4.3, 3.1.4.4 indicate that the two main pathways affected by genistein and daidzein are the estrogen and thyroid pathways and that the biological activity of daidzein is at least an order of magnitude lower than genistein. To investigate this further and to derive LOEC values [rather than IC_50_ values reported by others e.g., ER transactivation assays conducted by Kuiper et al. (1998)], both chemicals were tested in the EATS panel. This includes Chemical Activated Luciferase gene eXpression (CALUX^®^) ER, AR and TRβ transactivation assays, human thyroid peroxidase (hTPO) inhibition, Transthyretin (TTR)-binding and H295R steroidogenesis assays to investigate potential MoA(s) for reproductive toxicity (van der Burg et al., 2015; van Vugt-Lussenburg et al., 2018; Collet et al., 2019) (see Supplementary Materials S1 for assay details and methods). The outcomes of the EATS assays are summarized in Table 6.

**TABLE 6:** Summary of EATS results presented as a heatmap. The LOEC values are shown in Log M; the color indicates the potency (yellow < orange < red < purple). For comparison, the LOEC values of the individual reference compounds of the assays are shown in the rightmost column. The LOEC is defined as the lowest concentration where the compound significantly activates the assay, which is set to 10% of the maximum reference compound activity for agonist assays, and 20% of the maximum reference compound activity for antagonist assays.

	Genistein		Daidzein		Reference	
Assay	−S9	+S9	−S9	+S9	Value	Name
Cytotoxicity	>−5	>−5	>−5	>−5	−6.6	Tributyltin acetate
ERα receptor	−8.2	−8.3	−6.9	−7.0	−12.2	17β-estradiol
Anti-ERα antiestrogen	>−5	>−5	>−5	>−5	−8.3	Tamoxifen
AR androgens	>−5	>−5	>−5	>−5	−10.1	Dihydrotestosterone
Anti-AR anti androgens	>−5	>−5	>−5	>−5	−7.7	Flutamide
TRß thyroid	>−5	>−5	>−5	>−5	−9.9	T3 (triiodothyronine)
Anti-TRß antithyroid	>−5	>−5	> -5	> -5	−6.9	Deoxynivalenol
hTPO inhibition (thyroid)	−4.7	>−4.7	−4.6	>−4.7	−6.3	Methimazole
TTR binding (thyroid)	−6.7	−6.7	−6.0	−6.0	−7.9	Tetrabromobisphenol A
Steroidogenesis (ERα) 17β-estradiol	>−6	>−6.7	>−5.5	>−5.7	−6.0	Forskolin (↑), prochloraz (↓)
Steroidogenesis (AR) Testosterone	−5.5	−5.7	−6.0	−5.7	−6.0	Forskolin (↑), prochloraz (↓)

The cytotoxicity of genistein and daidzein was tested to ensure that the EATS assay outcomes were not impacted by cytotoxic effects (data not shown). Neither chemical was cytotoxic up to the highest concentration of 10 mM, in the absence or presence of rat liver S9. The rat liver S9 was incubated with cofactors that mediate phase 1 pathways (i.e., an NADPH and an NADPH-regenerating system); therefore, since genistein and daidzein are only conjugated via phase 2 pathways, the cytotoxicity was not expected to be altered by the inclusion of S9. This was confirmed in this study, since preincubation with liver S9 did not significantly influence any of the assay results.

The EATS-related assay results (log values are summarized in Table 6) identified genistein and daidzein as potent ligands for the estrogen-alpha (ERα) receptor. Daidzein was an order of magnitude less potent than genistein in activating the ERα CALUX assay (PC_50_ was 3.0 × 10^−8^ ± 1.4×10^−8^ M for genistein and 2.5 × 10^−7^ ± 0 M for daidzein). The LOEC was 6.5 × 10^−9^ ± 2.1×10^−9^ M for genistein and 1.1 × 10^−7^ ± 1.8×10^−8^ M for daidzein. While they exhibited estrogenic activity, they did not activate or antagonize the androgen receptor. Genistein and daidzein did not activate or antagonize the thyroid receptor-β; however, they were both shown to have potential thyroid effects, since they inhibited the binding of T_4_ to TTR and inhibited hTPO. The LOEC for inhibition of TTR binding by genistein was 2.1 × 10^−7^ ± 6.7 × 10^−8^, while daidzein was slightly less potent, with a LOEC of 1.0 × 10^−6^ ± 0. The LOECs for hTPO inhibition was 1.9 × 10^−5^ ± 8.9 × 10^−6^ M for genistein and 2.8 × 10^−5^ ± 1.3 × 10^−5^ M for daidzein. Neither genistein nor daidzein affected the production of estrogens up to the highest tested concentration of 1 × 10^−6^ M. However, androgen synthesis was greatly impaired, with LOECs of 3.2 × 10^−6^ ± 0 M for genistein and 2.1 × 10^−6^ ± 1.5 × 10^−6^ M for daidzein.

Comparison with the LOECs of each assay’s reference compound (rightmost column) shows that genistein and daidzein are 4-5 orders of magnitude less potent than the ERα reference compound, 17β-estradiol, and 1-2 orders of magnitude less potent than the reference compounds of the hTPO- and TTR binding assays, and approximately as potent as the testosterone synthesis inhibition reference compound. The lowest PoDs were based on the ERα + S9, for which the LOECs were 5.2 ± 2.1 nM for genistein and 100 ± 0 nM for daidzein. The presence of rat liver S9 mix did not influence the estrogenic potency of either chemical in this assay, whereby the LOECs for genistein and daidzein in incubations of ERα–S9 were 6.5 nM ± 2.1 nM and 110 nM ± 18 nM, respectively, which were not statistically significantly different from the LOEC + S9).

##### 3.3.1.2 Tier 2, step 7B: refinement of *in vitro* assays—biokinetics in HepG2 and MCF-7 cells

Nominal concentrations of test chemical were used to derive the PoDs for daidzein and genistein; however, these may differ from the actual concentrations present in the medium and in the cells themselves. Therefore, biokinetics experiments were conducted to determine the correlation between nominal and measured extracellular and intracellular concentrations of daidzein and genistein according to the conditions of the toxicogenomics experiments. Measurements were conducted in the target cell line, MCF-7 cells, and a representative hepatic cell line, HepG2 cells. The biokinetics measurements were not conducted in HepaRG cells; however, genistein and daidzein are reported to be extensively metabolized to sulfate and glucuronide conjugates (Tao T.-P. et al., 2023; Tao T. P. et al., 2023), which have lower bioactivities than the parent chemicals.

###### 3.3.1.2.1 Impact of well format on biokinetics

**FIGURE 6:**
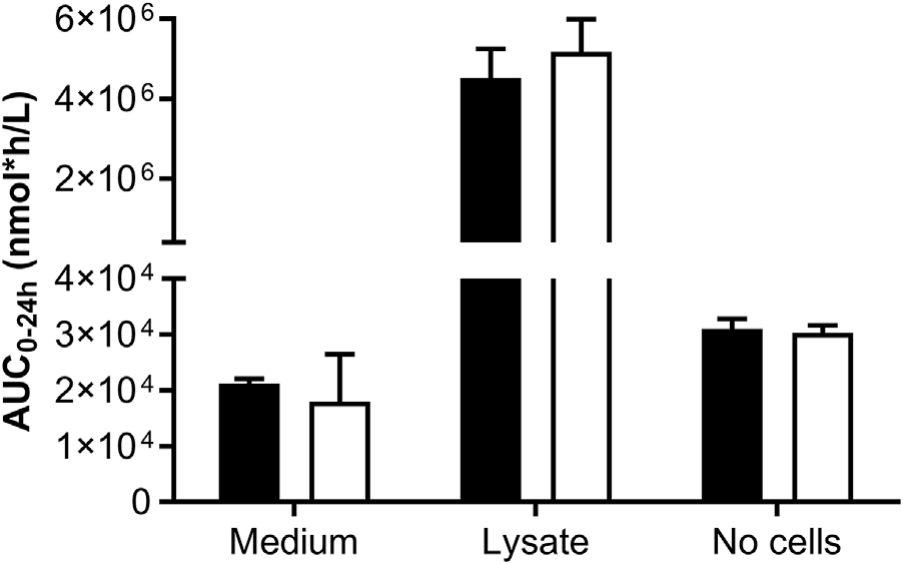
Comparison of the biokinetics of genistein in HepG2 cells measured in different well formats. The AUC was calculated from the concentration over time in the medium and lysate of HepG2 cells cultured in 48-well (black bars) and 96-well (white bars) format. The initial nominal concentration of genistein was 1.3 µM and the seeding density was 51,000 cells/well in 48-well plates and 17,000 cell/well in 96-well plates. Single wells from 48-well plates were analyzed and triplicate wells were pooled from 96-well plates. Control wells without cells were also included to measure chemical stability over time and potential non-specific binding to the wells.

Incubations for the toxicogenomics analyses were conducted in 384-well format plates; however, this format poses technical challenges when analyzing the concentrations of the test chemical and potential metabolites. The 48-well plates were the preferred culture format to achieve a good analytical sensitivity; therefore, two pilot studies were conducted to determine whether the biokinetics measurements were comparable across culture plate formats. In the first pilot experiment, the biokinetics of a single concentration of genistein in 48-well and 96-well plates was measured in one laboratory (Figure 6). This indicated that the overall exposure to the cells, according to the AUC_0–24 h_, was comparable using both well formats. This also indicated that the concentration of genistein associated with the cell lysate was much higher (millimolar) than the concentration in the medium or the nominal concentrations (micromolar). The incubations without cells indicated that genistein did not degrade, evaporate or bind to the plastic since the concentration at 0 h and 24 h were similar to the nominal concentrations (1.33 ± 0.06 µM and 1.26 ± 0.10 µM, respectively, in 48-well and 1.11 ± 0.06 µM and 1.41 ± 0.06 µM, respectively, in 96-well plates).

###### 3.3.1.2.2 Biokinetics of daidzein and genistein in HepG2 and MCF-7 cells

**FIGURE 7:**
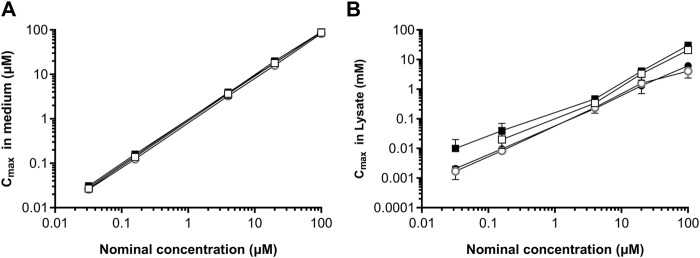
Comparison of maximum concentrations of genistein and daidzein in HepG2 and MCF-7 cells. C_max_ values were measured in the medium **(A)** and lysates **(B)** for genistein (open symbols) and daidzein (closed symbols) in HepG2 cells (squares) and MCF-7 cells (circles) cultured in 48-well format. Values are a mean ± SD, *n* = 3 wells per treatment.

The C_max_ concentrations of daidzein and genistein in the medium of HepG2 and MCF-7 cell incubations were similar to i.e., within 1.3-fold of the nominal concentrations (Figure 7A). When the volumes of the cells were incorporated into the calculation of the (free and bound) concentrations associated with the lysate, the resulting values were in the millimolar range (Figure 7B), thus over 1000-fold higher than the nominal concentrations. This indicates that a lack of response in the toxicogenomic assay is not due to a lack of intracellular exposure to the test chemicals. Moreover, the concentration at the site of action (genes) is at least 1000-fold higher than the nominal concentration. Therefore, the nominal concentration did not need to be adjusted for the NGRA as it represents a conservative value.

**FIGURE 8:**
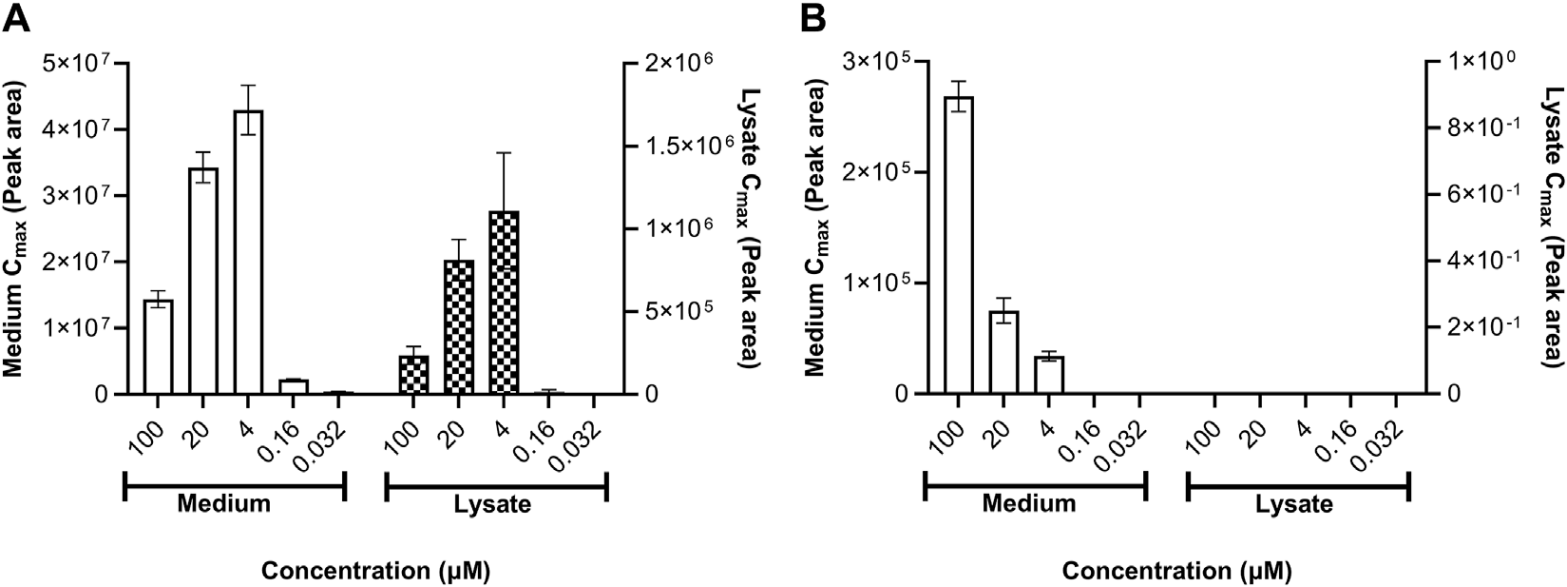
**(A)** Genistein sulfate formation in HepG2 and **(B)** genistein glucuronide formation in MCF-7 cells. The relative amounts of genistein-sulfate in HepG2 cells and genistein glucuronide in MCF-7 cells in the medium and lysate samples were semi-quantified according to the peak area (a reference standard for this metabolite was not available). Values are a mean ± SD, *n* = 3 wells.

Daidzein was not metabolized by HepG2 or MCF-7 cells (both of which are reported to exhibit low, if any, metabolizing activities (Falany and Falany, 1997; Hewitt and Hewitt, 2004). By contrast, genistein was metabolized by both cell types; however, it was sulfated by HepG2 cells and glucuronidated by MCF-7 cells (Figure 8). The formation of genistein-sulfate in HepG2 cells was linear over time (data not shown) but it was not concentration-dependent, such that the amount formed was lower at 100 and 20 µM than at 4 µM (Figure 8A). This indicates that, while no overt toxicity was observed according to lactate dehydrogenase leakage, these highest concentrations decreased the metabolic capacity of the cells (saturation of the enzymes would have led to a plateau and not a decrease). The formation of genistein glucuronide in MCF-7 cells was time- and concentration-dependent, indicating that these cells were not affected by cytotoxicity.

##### 3.3.1.3 Tier 2, step 7C: refinement of bioavailability for PBPK modeling

Additional information was required to determine whether an adjustment factor was needed to account for a difference in the absorption of daidzein after topical application and whether the absorption was impacted by the use of the intended (body lotion base) formulation. The transdermal absorption of genistein applied on the skin in olive oil has been studied *in vivo* by Vänttinen and Moravcova (2001). The excretion rate in urine and the concentration in plasma were significantly decreased after repeated transdermal application. The authors concluded that genistein may be captured in the skin following repeated transdermal application. We therefore conducted skin penetration and metabolism assays to determine the impact of (1) different doses after application in ethanol and (2) application in ethanol vs. body lotion formulation on the cutaneous distribution of genistein and daidzein in native human skin. The results of this study are described in detail by Géniès et al. (2024) and are summarized here.

The dermal bioavailability of 3, 10, and 30 nmol/cm^2^ genistein and daidzein after topical application in ethanol to fresh viable human native skin was high (>60% of the applied dose). There was a marked impact of the vehicle on the cutaneous distribution of genistein, such that the bioavailability was markedly decreased when it was applied in the formulation (e.g., from 62.6% ± 10.1% of the applied dose in ethanol to 12.7% ± 6.9% in formulation for 30 nmol/cm^2^ in formulation applied to fresh human skin). The impact of the formulation demonstrated for genistein was also observed for daidzein (e.g., from 59.8% ± 6.7% of the applied dose in ethanol to 7.3 ± 4.5 in formulation for 30 nmol/cm^2^ in formulation applied to fresh human skin) indeed, there was no statistical difference between the values for genistein and daidzein.

The bioavailability of ^14^C-labelled genistein (i.e., parent chemical and metabolites) applied at 3 nmol/cm^2^ in the body lotion formulation (equivalent to the expected dose applied in a cosmetic ingredient) was higher than that of daidzein (40.1% ± 8.8% and 24.7% ± 12.4% of the applied dose, respectively); however, the amount of parent chemical entering the systemic circulation was lower for genistein than daidzein (7.2% ± 15.1% and 13.5% ± 7.0% of the applied dose, respectively) due to the more extensive first-pass metabolism of genistein in the skin (after 24 h, 70%–90% of genistein was metabolized compared to 55% of daidzein). This is important because the parent chemicals are indicated to be bioactive, not their metabolites. Both chemicals were metabolized to sulfate and glucuronide conjugates, the ratios of which were the same regardless of the formulation.

The *ex vivo* absorption and metabolism experiments on frozen and fresh viable human skin were used to calibrate the dermal model in the human PBPK model [described by Najjar et al. (2024)]. For human exposure, the aim is to estimate a safe dose when used in a body lotion or face cream. The dermal absorption for genistein and daidzein was set at 40% and 25%, respectively. The *ex vivo* experiments also indicated that both chemicals were metabolized as they penetrate viable skin. When the simulations were run with and without skin metabolism, there was a difference of 3-fold in parent kinetics. Therefore, metabolism was considered by incorporating a model to characterize the rate of metabolism and then fitted it to the metabolism measured in *ex vivo* viable skin.

### 3.4 Step 8 perform a read-across to derive a PoD for daidzein

A summary of all the LOEC or No Observed Effect Concentration (NOEC) values for genistein and daidzein tested in the *in vitro* assays is shown in Table 7. According to the *in silico* and *in vitro* alerts, the main pathway affected by genistein and daidzein is the estrogen pathway. The lowest PoD for both chemicals was from the pharmacology profiling assays, in which the affinity of genistein to ERβ was higher than to ERα. However, binding of a ligand to a receptor does not automatically translate into a corresponding potency of a biological activity. For example, the fold induction of gene reporter activity by the reference estrogen, 17β-estradiol, was higher in HEK293 cells transfected with ERα than with ERβ (Kuiper et al., 1998). In the same study, the binding affinity of genistein to ERα and ERβ was measured, along with gene induction responses in the HEK293 transactivation assay. While the binding affinity of genistein to ERβ was higher than to ERα, it was equipotent in the HEK293 transactivation assay with cells transfected with ERα or ERβ (Kuiper et al., 1998). Thus, the EATS panel using ERα will detect most estrogenic chemicals that interact with ERβ. Therefore, the PoD in this safety assessment was based on biological functional activity (i.e., estrogenic activity), rather than the affinity of the interaction between a ligand and receptor. In support of this, the LOEC for genistein transactivation of ERα (6.5 nM) was lower than the LOEC for genistein binding to ERα in the pharmacology profiling assays (44 nM). In addition, ERα is reported to be more related to adversity than ERβ (SCCS, 2022). The lowest PoD for daidzein was based on the ERα + S9, for which the LOEC was 100 ± 0.0 nM. While this was the lowest PoD, the fact that it was the value in the presence of rat liver S9 mix does not mean that metabolic activation was required, since the LOEC for ERα without S9 of 110 nM ± 1.8 nM and was not statistically significantly different from the LOEC + S9.

**TABLE 7:** Summary of LOEC or BMD values for genistein and daidzein tested in *in vitro* assays.

*In vitro* assay	LOEC/NOEC	Endpoint	Daidzein	Genistein
Cell stress	LOEC	↓ in mitochondrial membrane potential	10.8 µM	11.6 µM
CALUX ERα	LOEC	Agonist	100 nM	6.5 nM
TPO	LOEC	Inhibition	28 µM	19 µM
TTR	LOEC	Inhibition of binding of T_4_ to TTR	1 µM	0.21 µM
AR	Not applicable	Agonist/antagonist	No effect	No effect
TRβ	Not applicable	Agonist/antagonist	No effect	No effect
Estrogen synthesis	Not applicable	Increase/decrease	No effect	No effect
Androgen synthesis	Not applicable	Increase/decrease	No effect	No effect
Pharmacology profiling: ERα	LOEC	Binding to receptor	35.2 nM	44.0 nM
Pharmacology profiling: ERβ	LOEC	Binding to receptor	3.2 nM	0.52 nM
Transcriptomics: MCF-7 cells	BMD/NOEC	Gene pathway deregulation	38 nM	6.5 nM
Transcriptomics: HepG2 cells	BMD/NOEC	Gene pathway deregulation	16.2 µM	15.7 µM
Transcriptomics: HepaRG cells	BMD/NOEC	Gene pathway deregulation	33.1 µM	40.0 nM

To support the selection of the ERα + S9 LOEC as the most relevant *in vitro* PoD for both chemicals, the concordance between the *in vitro* PoD and the predicted plasma concentration of the *in vivo* NOAEL of 0.3 mg/kg/day were compared [described by Najjar et al. (2024)]. The predicted mean total (C_max, total_) and unbound (C_max,u_) plasma concentrations of genistein were 24.1 nM and 0.48 nM, respectively. The *in vitro* LOEC for genistein derived from the CALUX^®^ ERα transactivation assay was 5.2 nM. To estimate the PoD i.e., NOEC, the LOEC was divided by a factor of 3 [recommended by Yang et al. (2017)], resulting in a NOEC of 1.73 nM. This *in vitro* NOEC was ∼14-fold lower than the equivalent *in vivo* NOAEL C_max, total_ (indicating the greater conservatism of the *in vitro* NGRA than the PoD used in a traditional risk assessment) but was in the same order of magnitude (nM range) as the C_max,fu_, indicting the PBPK model could predict relevant plasma concentrations. The *in vitro* NOEC of 1.73 nM was similar to the equivalent unbound plasma concentrations (mean and CI95 *in vivo* NOAEL C_max,u_ values were 0.48 and 1.37 nM, respectively). This indicates that the fraction unbound of genistein (the active form in blood) may represent the most relevant internal dose metrics to compare with the *in vitro* PoD for genistein and daidzein, whereas the total concentration can be considered to the most conservative value.

### 3.5 Step 9: NGRA for daidzein

#### 3.5.1 Step 9A: derivation of highest daidzein concentration in a body lotion

**FIGURE 9:**
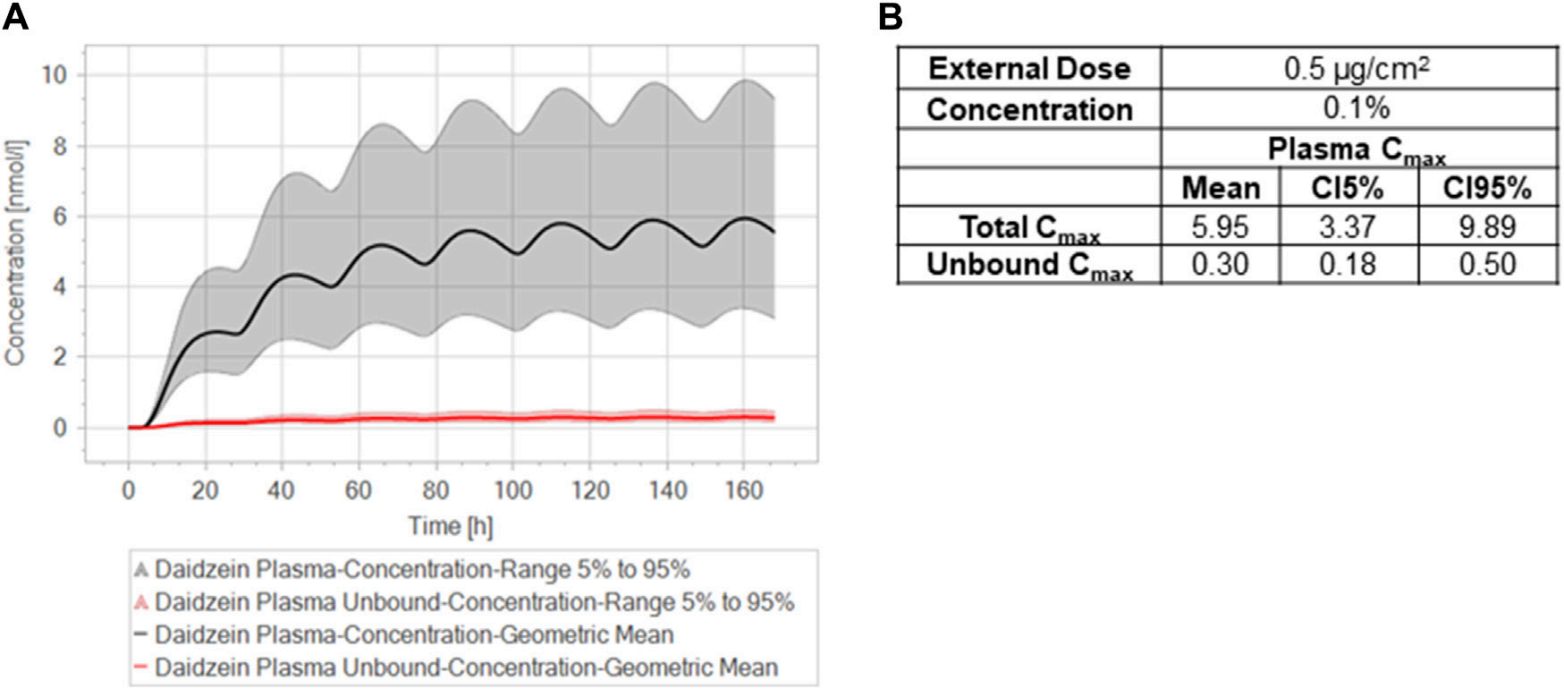
Estimation of a safe concentration of daidzein in a body lotion using the calibrated dermal module in the human PBPK model and the lowest PoD. **(A)** Simulation of target total (10 nM) and unbound plasma concentrations of daidzein after repeated dermal exposure of a body lotion. **(B)** Summary of total and unbound plasma concentrations of daidzein and calculation of the exposure dose of 0.5 μg/cm^2^ at 0.1% daidzein.

The NGRA aimed to derive the maximal safe concentration of daidzein for a body lotion exposure scenario following typical safety assessment factors to determine a safe margin of exposure. This concentration was estimated using the calibrated dermal module in the human PBPK model and the lowest PoD based on the CALUX ERα assay (Figure 9). A safety assessment factor of 3 was applied to convert the LOEC for the ERα identified in the CALUX assay (100 nM) into a NOEC estimation, resulting in an estimated NOEC of 33 nM. An additional safety factor of 3.3 was applied to account for intra-individual variability. The estimated safe plasma concentration of 10 nM was then extrapolated to the corresponding external dose by iteratively altering the simulated dosing scenario until the calculated plasma concentration was similar to 10 nM. The resulting external dose was estimated to be 0.5 μg/cm^2^ for a body lotion containing 0.1% daidzein. This is 5-fold higher than the concentration of daidzein considered safe by the SCCS for leave-on cosmetic products. However, the concentration of 0.02% considered and communicated as safe by the SCCS were not the maximum safe concentration but rather the concentration depicted in the SCCS mandate. Talsness et al. (2015) and Retana-Márquez et al. (2016) reported LOELs of 5 mg/kg in relevant studies for the oral and the subcutaneous route, respectively. The SCCS concluded that these LOELs could be considered as a NOAEL considering that the effects reported were not directly associated with fertility reduction in male rats. For a 0.02% daidzein body lotion, the SCCS calculated Margins of Safety based on PoDs from the oral and subcutaneous route of 96 and 385, respectively. These MoS were based on very conservative assumptions of 25% bioavailability for the oral route and a worst case bioavailability of 100% for the subcutaneous route compared to a conservative dermal penetration assumption of 50% due to a lack of information on the absorption and metabolism of genistein or daidzein in human skin. In the current study, an accurate measurement of absorption and metabolism was available, which showed that extensive first-pass metabolism to non-toxic metabolites occurred as the chemicals permeated the skin. The amount of parent chemical entering the systemic circulation was approximately 5-fold lower than the default value of 50% (7.2% ± 15.1% genistein and 13.5% ± 7.0% of the applied dose daidzein). Therefore, the difference in the absorbed amount used to calculate the systemic exposure dose accounted for the difference between the safe concentrations of daidzein derived by the NGRA and SCCS safety assessments.

#### 3.5.2 Step 9B: estimation of the *in vivo* NOAEL for daidzein using read-across

To investigate whether the difference between the NGRA and SCCS concentrations could have been due to uncertainties in the estimation of the PoD or the robustness of the PBPK model, the rat oral PBPK model was used to convert the *in vitro* PoD of 10 nM for daidzein to an external oral NOAEL in rats. The LOEC of 100 nM was converted to a NOEC by dividing by 3. No additional conversion factors were applied since this is a direct comparison without safety factors included. The resulting external NOAEL was estimated to be 4.1 mg/kg/day. This compared very well with the *in vivo* PoD of 5 mg/kg/day for daidzein, derived from a study showing some effects on male testes tissue, sperm count and testosterone levels but no indication for a reduction of fertility performances in rats (SCCS, 2022). This provides good evidence that the rat oral PBPK model provided accurate estimates of internal and external exposure and that the *in vitro* PoD was relevant in the safety assessment of daidzein.

#### 3.5.3 Step 9C: bioactivity exposure ratios (BERs) using SCCS recommended concentrations of daidzein

Another approach used to compare the NGRA and SCCS derived safe concentrations was to calculate bioactivity exposure ratios (BERs) using the *in vitro* PoD and the plasma concentrations after application of a body lotion containing 0.1% and 0.02% daidzein (Table 8). The concept of BER is mentioned in the SCCS Notes of Guidance (SCCS, 2023) and involves evaluating the relationship between the biological activity of a substance and its internal exposure level (Dent et al., 2019; Dent et al., 2021). These are analogous to the margin of exposure used in traditional risk assessments, whereby chemicals with a lower BER possess a higher potential for risk. If the BER is substantially greater than 1, it suggests that the estimated exposure is lower than the level at which adverse effects are observed, indicating a potentially acceptable safety margin. If the BER is close to or less than 1, it suggests the need to refine the assessment. These refinements could either be to increase the relevance of the exposure estimate, or further understanding whether the bioactivity seen is likely to result in an adverse health effect at those exposure levels.

**TABLE 8:** Bioactivity effect ratio (BER) values for estrogenic effects (the lowest PoD) after topical application of a body lotion containing 0.1% and 0.02% daidzein.

Bioactivity PoD (nM)	Exposure parameter	Body lotion with 0.1% daidzein		Body lotion with 0.02% daidzein	
		Exposure value	BER	Exposure value	BER
10	Mean C_max, total_ (nM)	5.95	10/5.95 = 1.7	1.18	10/1.18 = 8.5
10	C_max, total_ (CI5-95%) (nM)	3.37–9.89	10/3.37 = 3.0	0.68–1.96	10/0.68 = 14.7
			10/9.89 = 1.0		10/1.96 = 5.1
10	C_max,fu_ (nM)	0.3	10/0.3 = 33*	0.06	10/0.06 = 167^a^
10	C_max,fu_ (CI5-95%) (nM)	0.18–0.50	10/0.18 = 56*	0.04–0.10	10/0.04 = 250^a^
			10/0.50 = 20*		10/0.10 = 100^a^

^a^ The *in vitro* PoD was not adjusted for free concentration; therefore, this value is likely to be an overestimation.

When mean total plasma concentrations were used in the calculation of the BER, the values for 0.1% and 0.02% daidzein were greater than 1 and of a similar order of magnitude (1.7 and 8.5, respectively), indicating that the estimated exposure is lower than the level at which estrogenic or other biological effects are observed. When the most conservative estimation of internal exposure was considered i.e., the CI95% plasma C_max, total_, the BERs were still ≥1.

### 3.6 Step 10 assess level of confidence

The major assumptions and areas of resulting uncertainty in the risk assessment to determine the level of confidence and appropriate decision contexts and their impact on the assessment are listed in Table 9. The data and assay endpoints had a moderate to high confidence. There was high confidence in the EATS assay result and the PBPK model, which were both used to conclude on the safety of daidzein.

**TABLE 9:** Uncertainties in the NAMs used in the case study.

Data type/Endpoint	How used^a^	Assumptions	Level of confidence and/or uncertainty
ADME Properties	RA	Skin penetration and metabolism assay provides good quality data for use in refining the PBPK model	High confidence: Assay was conducted according to OECD test guidelines and SCCS basic criteria
CALUX assays/ER activity	RA	Assay provides good quality data for the target and source chemicals on the ER binding and activation. The assay provides a potency trend among target compound and positive control	High confidence: The assay was performed according to OECD TG by an experienced lab. CALUX assays are based using U2-OS cells, which have no endogenous receptors. This makes the assay highly specific and reduces the uncertainty. U2-OS cells have limited metabolic capacity, which might lead to false positive results if an active parent molecule would be readily metabolized *in vivo*. This uncertainty was reduced by performing the assays ± liver S9 extract
PBPK	RA	PBBK model will estimate internal exposure of the target chemical based on different external exposure scenarios. Models were used to calculate the internal exposure resulting from the intended consumer use scenario	High confidence: PBBK model was validated using *in vivo* genistein data. Internal exposure from the *in vivo* rat study was accurately predicted. The ability to rely on a measure of internal rather than external exposure reduces the uncertainty in the risk assessment by incorporating chemical-specific information on the ADME parameters of the chemical in the experimental animal and the human. The *in vitro* measurement of dermal absorption was used to calibrate the PBPK dermal module, thus decreasing uncertainty. The conversion of *in vitro* to *in vivo* doses and vice versa correlated well with the *in vivo* NOAELs and *in vitro* PoDs, respectively, thus providing a high confidence in the PBPK model
Consumer exposure (applied dose)	RA	Use in a body lotion is considered a reasonable worst-case scenario	Moderate confidence: Increases confidence that assessment is conservative
Molecular Docking/ER activity	WOE	These docking simulations can characterize the binding probability of parent and metabolites to 12 nuclear receptors	Moderate confidence: Docking simulations indicate that the metabolites of genistein are less likely to bind to the target receptors that the parent chemicals
ToxCast/Potency	WOE	ToxCast can inform on MoA and potency	Moderate confidence: Based on ToxCast ER activity assays relative potency scaling factors could be derived. Minor uncertainty remains regarding the coverage of ToxCast assays and metabolic capacity
Cell stress	WOE	Nontargeted approach used to indicate nonspecific toxicity leading to cell stress	Moderate confidence: Panel of assays is reported to provide data which are protective of consumer health
Pharmacology profiling	WOE	Provides an indication of possible targets of interest and increases biological coverage	Moderate confidence: Although assay indicates additional targets, the binding to a receptor does not directly correlate to a bioactivity potency, e.g., ERα and ERβ binding and estrogenic activity
Toxicogenomics	WOE	Toxicogenomic data can inform on the gene changes and support the identification of the specific biologic activity of genistein and daidzein	Moderate confidence: The toxicogenomics studies were conducted under standardized conditions and genes analysed using a validated commercial transcriptional profiling platform and statistical data analysis packages. There is uncertainty regarding biological coverage, although 3 cell lines were used

^a^ How data was used in the case: RA, risk assessment; WOE, weight of evidence for biological effects.

## 4 Summary and conclusion

This case study demonstrates the application of the 10-step read-across framework described by Alexander-White et al. (2022) for use in cases where a TTC approach to cosmetics safety assessment is not possible. The tiered workflow used herein (for which the methods are summarized in Figure 1 and the results summarized in Table 10) describes the justification and use of data for the source chemical, genistein, in a read-across strategy to fill the endpoint data gap for the target chemical, daidzein. This case study describes the NGRA of daidzein present in a leave-on cosmetic product, with the aim of estimating the highest concentration of daidzein that can be used safely in a body lotion. An exposure-led approach was taken to extrapolate the lowest relevant *in vitro* bioactivity PoD, assessed using multiple biomarkers and covering a diverse biological space, to an estimated external concentration in humans.

**TABLE 10:** Summary of results and their interpretation for the NGRA.

Tier level	Result
Tier 0: Identify use scenario and suitable analogue for read-across	
Step 1: Identification of use scenario of target chemical	Repeated topical application of daidzein as a body lotion. The aim of the case study was to identify the highest concentration of daidzein that can be safely used in the formulation
Step 2: Identify molecular structure of target chemical and its metabolites	It is a member of the class of 7-hydroxyisoflavones, whereby 7-hydroxyisoflavone is substituted by an additional hydroxy group at position 4'. Major metabolites were predicted to be glucuronide and sulfate conjugates, which were confirmed in *in vitro* assays
Step 3: Collate supporting data on target chemical and its metabolites and define data gaps	Two main assumptions: (1) daidzein was assumed to be a new cosmetic ingredient; (2) all *in vitro* and legacy *in vivo* data for genistein were considered. This meant that there were no *in vivo* pharmacokinetics or toxicodynamics data for daidzein. While *in vitro* data for daidzein were available in the literature, new data to broaden the biological space were needed
Step 4: Analogue identification, existing data and determine similarity hypothesis	Genistein was predicted to be the most suitable analogue for daidzein. The structures of the target and source chemicals are very similar, with the only difference being an additional hydroxyl group on genistein compared to daidzein. Read-across based on high similarity in chemical structures, metabolites and physicochemical properties to substantiate the suitability of genistein as the source comparator for daidzein
	*In silico* models predicted genistein to be conjugated to glucuronide and sulfate conjugates, which were detected in several *in vitro* MetID assays
	*In vivo* legacy data for genistein were evaluated, from which a multi-generation study was selected for the PoD. The lowest NOAEL of 0.3 mg/kg (5 ppm) in male rats
	*In silico* and *in vitro* toxicodynamic data indicate that the two main pathways affected are the estrogen and thyroid pathways and that the biological activity of daidzein is at least an order of magnitude lower than genistein. The profilers highlighted as relevant for reproductive toxicity i.e., the DART scheme, ER binding, Retinoic Acid Receptor binding and the rtER Expert System from US EPA, showed that genistein and daidzein were similar with respect to DART and ER binding properties. None of the metabolites were positive in the molecular docking tool for ED – only the parent chemical, genistein, was positive
Tier 1: Systemic bioavailability and ADME properties of analogue and target chemicals	
Step 5: Systemic bioavailability and ADME properties to estimate internal concentrations of target and analogue chemicals	Main *in vitro* ADME properties of the two chemicals relating to systemic metabolism and clearance after oral application are similar. To provide an estimation of the *in vitro* PoD for the bioactivity assays, a PBPK model was built to convert the external NOAEL dose of genistein to an internal plasma concentration. The mean C_max, total_ and C_max,fu_ were estimated at 24.1 and 0.48 nM, respectively. These values were used to set the doses for the toxicogenomics and cell stress assays
Step 6: Supporting a similar MoA hypothesis	Neither chemical caused marked responses in cell stress assays
	In the 83-assay pharmacology profiling panel, there were only 11 hits for both chemicals. Daidzein was less potent than genistein and the ERα and ERβ were identified as the most potently affected hits. The LOECs based on the ERα were 44 nM for genistein and 35 nM for daidzein; however, the most potent effect was observed for the agonist binding to ERβ, with LOEC concentrations of 0.518 nM and 3.25 nM, respectively
	Transcriptomics analyses in MCF-7, HepG2 and HepaRG cells were used to inform on the broad biological activity and relative potency of genistein and daidzein. There are 71 genes in MCF-7 cells deregulated by both chemicals. The lowest median BMDs for daidzein and genistein were 38 nM and 6.5 nM, respectively (both in MCF-7 cells)
Tier 2: Application of read across approach	
Step 7A: Targeted testing to strengthen hypothesis and biokinetics	Genistein and daidzein were tested in EATS panel of CALUX^®^ ER, AR and TRβ transactivation, hTPO inhibition, TTR-binding and H295R steroidogenesis assays to investigate potential MoA(s) for reproductive toxicity The lowest LOECs were for ERα binding and were 5.2 ± 2.1 nM for genistein and 100 ± 0.0 nM for daidzein
Step 7B: Refinement of *in vitro* assays - biokinetics in HepG2 and MCF-7 cells	C_max_ concentrations of daidzein and genistein in the medium of HepG2 and MCF-7 cell incubations were similar to nominal concentrations. No impact of different well-formats on intracellular exposure
Step 7C: Refinement of bioavailability for PBPK modeling - skin penetration and metabolism data	*In vitro* skin penetration and metabolism assays using fresh and frozen human skin explants showed the bioavailability of the chemicals was comparable. The impact of the dose and formulation on the cutaneous distribution of both compounds was comparable; therefore, no adjustment was needed for the read-across regarding these aspects. The bioavailable amount of parent and metabolites was ∼40% for genistein and 25% for daidzein. Genistein and daidzein were both extensively metabolized by human native skin to sulfate and glucuronide conjugates (44%–74% and 21%–37% of the applied dose for 3–30 nmol/cm^2^ genistein and daidzein, respectively). These data were used to calibrate the dermal model in the human PBPK model. The PBPK model was used to convert the PoD in in vitro assays to an external safe dose
NGRA for daidzein	
Step 8: NGRA: Perform a read-across to derive a PoD for daidzein	The lowest PoD for daidzein was based on the ERα + S9, for which the LOEC was 100 ± 0.0 nM. The LOEC for the ERα in the CALUX assay (100 nM) was divided by 3 to estimate a NOEC of 33 nM and then a safety factor of 3.3 was applied to account for intra-individual variability (resulting in a plasma concentration value of 10 nM)
Step 9: NGRA: Extrapolation to a safe dose; estimation of *in vivo* NOAEL for daidzein; and calculation of BERs	The estimated safe plasma concentration of 10 nM was extrapolated to the corresponding external dose of 0.5 μg/cm^2^ for a body lotion and face cream. This equates to a concentration of 0.1%
	When *in vitro* PoD of 33 nM for daidzein was converted to an external oral NOAEL in rats, the value correlated with the *in vivo* NOAEL. This provides further evidence that the rat oral PBPK model provided accurate estimates of internal and external exposure and that the *in vitro* PoD was relevant in the safety assessment of both chemicals
	When mean C_max, total_ was used to calculate the BER for 0.1% and 0.02% daidzein, values were >1 and of a similar order of magnitude. These indicate a sufficient difference between concentrations causing bioactivity and estimated internal exposure to daidzein
Step 10: Assess level of confidence	The major assumptions and areas of resulting uncertainty in the risk assessment to determine the level of confidence and appropriate decision contexts and their impact on the assessment were highlighted

The key aspects of the hypothesis in this case study assumed (1) the same or similar MoAs are responsible for the observed effects; (2) the parent chemicals are metabolized by UGTs and SULTs in the skin or systemically after absorption, with glucuronide and/or sulfate conjugates being formed and (3) findings from available legacy *in vivo* studies for genistein, together with NAMs representing these mechanisms/pathways, would provide a weight of evidence that data from genistein can be used in a read-across to fill the theoretical toxicity data gap for daidzein. Furthermore, it was assumed that NAM data would provide evidence of a potency difference between genistein and daidzein, and that relative potency information could be used to inform the read-across safety assessment.

Several *in silico* and *in vitro* assays were used to evaluate the bioactivity of daidzein and genistein. The *in silico* models used to predict potential metabolites (GloryX and Meteor nexus) and potential targets (OECD QSAR Toolbox, Endocrine Disruptome tool) were consistent with each other and with the results from *in vitro* assays. The NAMs all indicated that the major effects of both chemicals were related to endocrine disruption, however, they also considered other targets by using assays which cover a broad biological space i.e., the cell stress panel, pharmacology profiling and transcriptional profiling. The NOECs relating to non-EATS effects derived from the cell-based assays, (i.e., cell stress panel and transcriptional profiling assays), were not lower than the PoD for estrogenic effects, indicating that the overall safety assessment should be based on this endpoint. Notably, LOEC value for daidzein binding to the ER in the pharmacology profiling assays was lower than the LOEC for its estrogenic effects in the CALUX assay. Moreover, the LOEC for genistein for ERα was lower in the CALUX than in the pharmacology profiling assay. Therefore, the translation of the binding potency from this assay is not directly proportional to a biological activity, in this case estrogenicity in a cell model. Therefore, results from pharmacology profiling assays can be used to flag potential targets but not necessarily to define a biological potency.

*In vitro* biokinetics assays have been used by others to refine the safety assessment e.g., phenoxyethanol (Hewitt et al., 2022). For the NGRA of phenoxyethanol, the major metabolite, phenoxyacetic acid, was identified as the most relevant in driving the assessment since its concentration in the kidney was predicted to far exceed that of phenoxyethanol in blood or other tissues. The *in vitro* intracellular concentrations (according to AUC_24_ values) in cells used in toxicity assays at the *in vitro* PoDs were compared with predicted *in vivo* tissue levels to conclude on the safety margin. In the current case study, *in vitro* biokinetics assays in HepG2 and MCF-7 cells were conducted to determine (1) whether the plate format impacted the exposure of the cells to the test chemicals, (2) whether either chemical was metabolized and (3) the concentrations associated with the medium and cell lysates. There was no impact of the well-format on the biokinetics of either chemical, indicating that exposure to cells was independent of the plate format. There was little (genistein) or no (daidzein) metabolism of the chemicals over 24 h; therefore, the gene changes measured in the transcriptomics assays could be considered to be due to the parent chemical and a worst-case scenario (since metabolism was considered to be a detoxification pathway). A limitation of the assays was that the metabolism in HepaRG cells was not measured; however, this was considered not to impact the safety assessment since the PoDs from the transcriptomics assays were not used in the NGRA. The *in vitro* biokinetics assays also indicated that the concentration of genistein associated with the cell lysate was much higher (millimolar) than the concentration in the medium or the nominal concentrations (micromolar). While this indicates potential accumulation in the cells, it is likely that the free concentrations of both chemicals are in equilibrium between the medium and intracellular space.

PBPK modelling was a central part of this case study. It was used to simulate 4 different exposure scenarios and these values helped to support the selection of the PoD. A luxury of a read-across NGRA is that the PBPK model can be validated using *in vivo* data for the source chemical (which is not a possibility with an *ab initio* case study and raises issues on its own in how to achieve validation). While the PBPK rat oral model was validated using *in vivo* rat pharmacokinetics data (generated prior to the testing ban), the human model was indirectly qualified because it was used to confirm external and internal concentrations which were correlated with SCCS approved concentration and *in vitro* PoDs, respectively. Human plasma concentrations, including inter-individual variability, were estimated using a PBPK model which was refined using measured human skin penetration and metabolism data. An important aspect of the PBPK model was that it incorporated extensive first-pass metabolism of genistein in the skin, which significantly decreases the internal exposure to this chemical when applied topically. The PBPK model also helped to support the relevance of the PoD selected for the NGRA by converting the *in vivo* NOAEL of genistein in rats to a plasma concentration which was comparable with the genistein NOEC based on ER agonism in the CALUX assay. Reverse-dosimetry of the *in vitro* PoD (also identified from the CALUX^®^ ERα transactivation assay) was also conducted to estimate the relevant external exposure to daidzein in a relevant cosmetic formulation. The LOEC for daidzein in the ERα transactivation assay was converted using reverse-dosimetry to an estimated external *in vivo* PoD of 4.1 mg/kg, which is in accordance with the known lower potency of daidzein compared to genistein (SCCS, 2022).

Although this case study aimed to derive the highest concentration of daidzein which can be safely used by consumers, rather than to determine a margin of safety to support a particular use concentration of the ingredient, it is interesting to compare the BERs derived using estimated plasma concentrations after topical application in a body lotion with those derived using reported measured concentrations of genistein following dietary intake. Daidzein is present in soy-derived foods and is consumed in large amounts in Asian countries. The consumption of soya products, and thus plasma concentrations of daidzein, varies across individuals but most strikingly, across populations. When the highest measured plasma concentrations of daidzein e.g., 3.14 µM, are compared with the NAM-derived PoD (10 nM), the resulting BER (10 nM/3.14 µM = 0.003) is at least 2 orders of magnitude lower than 1. This suggests that in theory, there may be a potential concern for adverse effects at internal exposure levels resulting from dietary intake. Clearly, this is not the case and moreover, dietary genistein is known to have beneficial effects.

When the results of this safety assessment were compared with the conclusion from a safety assessment using traditional methods, the NAM-based safety assessment appears to be sufficiently protective for the consumer. In the recent SCCS opinion on genistein (SCCS, 2022), the Margin of Safety (MoS) for daidzein applied at a concentration of 0.02% was calculated to be 96 (based on an oral PoD). The BERs derived using total plasma concentrations in this case study ranged between 5.1 and 14.7 and are lower than the MoS derived using animal data. This implies that the NGRA approach presented here is more conservative than the traditional risk assessment. This corroborates other analyses that have shown that NAM derived PoDs are often more conservative than animal-derived PoDs (Paul Friedman et al., 2020). If the unbound plasma concentrations are used for the NGRA and BERs of 100–250 are taken, the assessment is as conservative as the traditional risk assessment.

## Data Availability

The raw data supporting the conclusions of this article will be made available by the authors, without undue reservation.

## Abbreviations

ADME, Absorption, distribution, metabolism, and excretion; AR, Androgen receptor; BMD, Benchmark dose; CALUX, Chemical Activated Luciferase gene eXpression; Cmax, Maximum plasma concentration; CTD, Comparative Toxicogenomics Database; DART, Developmental And Reproductive Toxicology; EATS, Estrogen-, androgen-, thyroid signaling and steroidogenesis; ER, Estrogen receptor; Fup, Fraction unbound in the plasma; human thyroid peroxidase, (hTPO); ICCR, International Cooperation on Cosmetics Regulation; LOEL, Lowest observed effect concentration; LogP, Logarithm of octanol/water partition coefficient; LRSS, Long Range Science Strategy; MCF7, Breast cancer cell line; MEC, minimum effective concentration; MoA, Mode of action; MoS, Margin of Safety; NAM, New Approach Methodology; NGRA, Next-Generation Risk Assessment; NOAEL, No observed adverse effect level; PBPK, Physiologically-based pharmacokinetic modelling; PoD, Point of departure; QSAR, Quantitative structure–activity relationship; SCCS, Scientific Committee for Consumer Safety; TTR, Transthyretin; TTC, Threshold of Toxicological Concern; US EPA, United States Environmental Protection Agency.
