# Supplementary material for: Next-generation risk assessment read-across case study: application of a 10-step framework to derive a safe concentration of daidzein in a body lotion
Source: Front Pharmacol. 2024 Jun 19;15:1421601. doi: 10.3389/fphar.2024.1421601 (PMC11220827; doi:10.3389/fphar.2024.1421601)
Supplement: Supplementary file 1 [file DataSheet1.PDF]

Supplementary materials

Supplementary materials S1: Materials and Methods

S1.1. Materials

Daidzein (product code D7802) was from Sigma Aldrich and genistein (product number AG-CN2-0427) was from AdipoGen. All chemicals were of the highest purity. The body lotion formulation is a proprietary base formulation from Beiersdorf AG. The final preparations of genistein and daidzein were prepared at Pierre Fabre, Toulouse, France.

S1.2. *In silico* predictions

In silico predictions of metabolites were performed using Meteor Nexus version 3.1.0 (Lhasa Ltd.) using "Site of Metabolism Scoring (with Molecular Mass Variance)" with a relative threshold of 70 as reasoning methodology (default setting).

The source chemical for daidzein was predicted using ToxGPS version 4.

The Comparative Toxicogenomics Database (CTD, https://ctdbase.org).

Open-source prediction software (from the university of Hamburg https://software.zbh.uni-hamburg.de/) was used to predict the likely metabolites of genistein and daidzein.

The US EPA QSAR prediction model, Opera (https://github.com/kmansouri/OPERA.git), was used to predict the binding, agonism or antagonism of ER and AR by genistein and daidzein.

S1.3. Biokinetics in HepG2 and MCF-7 cells

In the pilot study, an initial nominal concentration of 1.3 µM genistein was incubated with HepG2 cells (from ECACC Salisbury, UK, Cat. No. 86012803) cultured in 48-well and 96-well plates for 24 h. The seeding density was 51,000 cells/well in 0.5 ml medium in 48-well plates and 17,000 cell/well in 96-well plates. The medium for HepG2 cells was Roswell Park Memorial Institute (RPMI) medium supplemented with 10% FBS, 2 mM L-GlutaMAX, 53 U/mL penicillin and 53 µg/mL streptomycin. The concentrations of genistein and its metabolites were measured over time in the medium and lysate samples. Samples from three 96-wells were pooled for analysis (to result in a total of 51,000 cells in a single sample), whereas single wells were analyzed from 48-well plates. Samples were precipitated with an equal volume of 100% acetonitrile before analysis. Triplicates were analyzed (i.e. 3 wells for 48-well plates and a total of 3 x 3 = 9 wells for 96-well plates.

In the main study, 5 concentrations of daidzein and genistein (0.032, 0.16, 4, 20 and 100 µM) were incubated in triplicate with HepG2 cells and MCF-7 cells for 24 h. The medium for MCF-7 cells was Minimal Essential Medium (EMEM) supplemented with 10% FBS, 2 mM L-GlutaMAX, 1% non-essential amino acids (NEAA), 53 U/mL penicillin and 53 µg/mL streptomycin. Medium and lysate samples were analyzed at 0, 1, 3, 6 and 24 h. The seeding density for both cell types was 51,000 cells/well in 48-well plates.

For pilot and main experiments, control wells without cells were also included to measure potential non-specific binding to the wells. The AUC values for the medium and lysates were calculated according to Hewitt et al. (2022).

For pilot and main experiments, the metabolic function of the cells was determined by incubating the cells with 5 µM ethoxycoumarin (dissolved in 100% acetonitrile) for 24 h. The metabolites were analyzed by high-performance liquid chromatography (HPLC)-HRMS (Q-Orbitrap) by means of accurate mass and MS/HRMS fragmentation for accurate mass structural analysis.

S1.4. LC-HRMS analysis of genistein and daidzein and its metabolites

Samples were analyzed for genistein and daidzein using the LC/MS Q-Exactive Plus (Thermo Scientific) instrument in both positive and negative ionization mode with high resolution (70,000) and accurate mass detection (Orbitrap™), according to (Tao et al. 2023). The MS was operated in the full scan MS-SIM (m/z: 50-750) mode. The m/z values for the [M-H]- ion of genistein and genistein glucuronide were 269.0453 and 445.0774, respectively. The m/z values for the [M-H]- ion of daidzein, daidzein glucuronide and daidzein sulfate were 253.0501, 431.0982 and 333.0071, respectively. The m/z values for the [M+H]+ ion of MMI was 115.0327. Diazepam was used as ISTD in the positive full scan mode. The specified limit of quantification (LOQ, defined as the concentrations where signal intensity reached 5 times the noise) for genistein, daidzein and MMI were 10, 5 and 15 nM, respectively.

S1.5. Cell stress assays

Genistein and daidzein were tested in 13 Multiparameter High Content Screening (HCS) assays: Mitochondrial/ Oxidative stress, GSH ROS MMP ATP & LDH assay, Oxidative stress assay (SRXN1 & NRF2), DNA damage (pH2Ax & p53), PLD & Steatosis assay, ER Stress (2 plate assay), Osmotic stress, Metal stress, Hypoxia/Inflammation, Necrosis, apoptosis and cell cycle arrest assay with inflammation markers, AhR translocation, Mitochondrial Potential (TMRE), Functional Mitochondrial Toxicity Assay: Seahorse Mitochondrial Profiling in HepG2 cells at 24 h. These targets have been shown to be predictive for compounds causing various forms of toxicity.

Experimental Procedure

HepG2 cells were plated on tissue culture treated plates in appropriate media at 37°C in 5 % CO_2 for 24 h prior to dosing of the cells. Test compounds were diluted in DMSO and serial dilutions were made in 0.5 % DMSO in growth media. Test compounds at 8 concentrations (300, 75, 18.75, 4.69, 1.17, 0.29, 0.073, 0.018 µM) in duplicates were then incubated for 24 h. Appropriate controls were included or each multiparameter HCS assay. At the end of the incubation period, the cells were loaded with the relevant dye/antibody for the appropriate time for each cell health marker. The plate was then scanned using an automated fluorescent cellular imager, ArrayScan® VTI or XTI (Thermo Scientific Cellomics). The plate designs provide simultaneous measurement of multiple cell health parameters for each of the 13 multiparameter HCS assays: Mitochondrial/ Oxidative stress, GSH, ROS, MMP, ATP & LDH assay, Oxidative stress assay (SRXN1 & NRF2), DNA damage (pH2Ax & p53), PLD & Steatosis assay, ER Stress (2 plate assay), Osmotic stress, Metal stress, Hypoxia/Inflammation,

Necrosis, apoptosis and cell cycle arrest assay with inflammation markers, AhR translocation. Mitochondrial Potential (TMRE), Functional Mitochondrial Toxicity Assay: Seahorse Mitochondrial Profiling. These targets have been selected to be predictive for compounds causing various forms of toxicity.

Data Analysis

For readouts other than cell viability, a statistical approach to data analysis was used. The vehicle controls were used to determine the definitions of "normal" for each parameter, then the software calculates the percentage of cells that are low or high responders (depending on the biological significance of a particular readout). The vehicle control wells were then used to determine significance limits for wells that have a greater than expected fraction of low or high responders. The minimum effective concentration (MEC) is determined from the lowest concentration whose mean value exceeds the significance level, provided either a clear dose-response relationship is observed, or at least two consecutive concentration points are above the significance level. AC_{50} values were also determined provided a clear dose-response relationship is observed.

S1.6. EATS assays

Cytotoxicity

Before analysis on the various bioassays, the cytotoxicity of genistein and daidzein was assessed using the U2-OS based CALUX cytotox bioassay. The cytotoxicity of each test chemical was assessed using the U2-OS based cytotoxicity CALUX bioassay. Concentrations causing >20% reduction of luminescence were considered cytotoxic. Only dilutions that did not show any signs of cytotoxicity (relative induction in the cytotoxicity CALUX bioassay > 80%) were used for final evaluation of analysis results. Only dilutions that did not show any signs of cytotoxicity (relative induction in the cytotox CALUX bioassay > 80%) were used for final evaluation of analysis results. All analysis results were expressed in molar (M) final in well.

CALUX bioassays

For determination of the (anti-)ERα, (anti-)AR and (anti-)TRβ CALUX activities, CALUX cells were seeded in assay medium. Following exposure of the CALUX cells to serial dilutions of the compounds in triplicate for 24 h, the induction of luciferase production was quantified by measuring luminescence following addition of the substrate luciferin. For antagonist assays, a fixed concentration of assay agonist was present in each well, to generate a basal signal. On each plate, a complete calibration curve for each respective bioassay was also analysed using the relevant reference compounds. For the analyses in the presence of rat liver S9, 0.03% (v/v) of PB/BNF induced rat liver S9 mix (including NADPH and an NADPH-regenerating system) was added to the cells directly after addition of the compounds, which remained there for the duration of the exposure period (24 h) (van Vugt-Lussenburg et al. 2018).

hTPO inhibition assay

hTPO was derived from Nthy-ori 3-1 cells. Cell lysate containing hTPO in Glycine-NaOH buffer (pH 9.0) was incubated for 30 minutes at 37°C in the presence of serial dilutions of the compounds in

DMSO (1% compound stock in incubation mixture). The incubation mixture was transferred to 96-well microtiter plates after which luminol (34.8 µM) and H_2O_2 (1.7 mM) were added. Luminescence was measured on a Berthold luminometer.

TTR-binding assay

Serial dilutions of the compounds were incubated in Tris-buffer (pH 8.0) overnight at 4°C in the presence of TTR (0.058 µM) and a fixed concentration of T4 (0.052 µM) (3.2% compound stock in incubation mixture). After incubation, TTR-bound and free T4 were separated on a Bio-Gel P-6DG column. The eluate was added to assay medium after which TRβ CALUX cells were exposed for 24 h (see CALUX bioassays). For this TRβ CALUX exposure, serum-free assay medium was used.

H295R steroidogenesis assay

To determine the effect of the compounds on sex hormone synthesis, H295R cells were seeded in 48 wells plates in assay medium, and exposed for 48h to a serial dilution of the compound in triplicate (0.1% v/v). To quantify the levels of oestrogens and androgens produced by the H295R cells after exposure, the assay medium was analysed on the ERα and AR CALUX bioassays as described previously (CALUX bioassays). Changes in hormone levels compared to a vehicle control exposure indicate that certain enzymes involved in steroidogenesis were being affected by the test compound.

Preincubation with rat liver S9 fraction

In contrast to the CALUX assays, the hTPO inhibition, TTR-TRβ and H295R assays are not directly compatible with rat liver S9 mix. To assess the effect of metabolism on the activity of the test compound for these assays, preincubations with rat liver S9 were performed. Compounds (10 µl DMSO stock solution) were incubated for 0 h (control) or 3 h (test condition) in a total volume of 1 ml TRIS buffer in the presence of 1% (v/v) PB/BNF induced rat liver S9 mix (including NADPH and an NADPH-regenerating system). After incubation, the reaction was stopped by the addition of ice cold acetone, and samples were frozen to stimulate protein precipitation. After thawing, the precipitated protein fraction was subsequently removed by centrifugation. The supernatant, containing the metabolites and any remaining non-metabolised parent compound, was concentrated under a gentle nitrogen flow. After evaporating to dryness, the sample was taken up in 50 µl DMSO followed by the addition of 50 µl TRIS buffer. This extract was used for the exposure of the hTPO inhibition, TTR-TRβ and H295R assays according to the usual protocol, at 2% (hTPO inhibition), 6.4% (TTR-TRβ) or 0.2% (H295R) exposure (v/v).

As a consequence, for these assays three different analyses were performed: analysis of the 'pure' compound dissolved in DMSO, analysis of the extract of the control preincubation 't=0', and analysis of the extract of the metabolized sample 't=3'. Because the sample work-up after preincubation may have a recovery of < 100%, the 'pure compound' analysis cannot be directly compared to the preincubated sample; therefore, the analysis of the pure compound is used to determine the potency of the compound on these three assays, while the t=0 vs t=3 preincubated samples are compared only with each other to assess if the compound's activity is influenced by the rat liver S9 metabolic fraction. In the TPO assay only, a slightly decreased signal was consistently observed in the solvent control t=3 preincubations; all the sample t=3 preincubations were therefore corrected for this decreased signal.

Data analysis

Relative light units (RLUs) of the CALUX assays were analysed as follows. For agonist assays, background values (exposure to DMSO only) were subtracted, and the maximum activity of the assay's reference compound was set to 100%. For antagonist assays, the basal signal (exposure to fixed concentration of agonist only) was set to 100%. Activity of the samples was scaled accordingly, resulting in expression of the data as "percentage of maximum reference response". Graphs were generated using the statistical software package GraphPad Prism V5.03. Using these graphs, lowest effect concentrations (LOECs) and PC50 concentrations were determined; LOEC is defined as the lowest concentration where the compound significantly activates the assay, which is set to 10% of the maximum reference compound activity for agonist assays, and 20% of the maximum reference compound activity for antagonist assays. The PC50 is defined as the concentration where the activity of the sample equals 50% of the maximum activity of the assay's reference compound.

For the H295R assay, the amount of estrogens and androgens produced by the H295R cells exposed to the test compound was divided by the amount of estrogens and androgens produced by the solvent control-exposed cells. A sample was considered 'active' if it decreased the hormone production 0.8-fold, or increased the hormone production 1.2-fold.

S1.7. Pharmacology profiling assays

Genistein and daidzein were tested at 10 µM in a panel of 83 assays for a range of pharmacological targets. The list of targets included the original panel of 44 targets (including receptors, ion channels, transporters and enzymes) recommended by 4 major pharmaceutical companies as significant liabilities in drug development (Bowes et al. 2012), together with additional targets that have been reasoned to link to pathological effects. Results showing an inhibition or stimulation higher than 50% are considered to represent significant effects (i.e. a "hit"). Inhibition (or stimulation) between 25% and 50% are indicative of weak to moderate effects and results showing an inhibition (or stimulation) lower than 25% are not considered significant and mostly attributable to variability of the signal around the control level. For several targets, additional concentration effect assays were conducted to determine the potency (IC_{50}). The concentrations tested (in duplicate) ranged between 30 nM and 100 µM.

Supplementary Table S4 Pharmacology profiling assay panel.

ASSAY NAME
5-HT transporter (h) (antagonist radioligand)
5-HT1A (h) (agonist radioligand)
5-HT2A (h) (agonist radioligand)
5-HT2B (h) (agonist radioligand)
5-HT3 (h) (antagonist radioligand)
A1 (h) (antagonist radioligand)
A2A (h) (agonist radioligand)
ACE (h)
ACE-2 (h)
acetylcholinesterase (h)
alpha 1A (h) (antagonist radioligand)

alpha 2A (h) (antagonist radioligand)
alpha 2B (h) (antagonist radioligand)
AMPA (agonist radioligand)
AR(h) (agonist radioligand)
Aromatase / CYP19A1
AT1 (h) (antagonist radioligand)
ATPase (Na+/K+)
beta 1 (h) (agonist radioligand)
beta 2 (h) (antagonist radioligand)
BZD (central)(h) (agonist radioligand)
Ca2+ channel (L, dihydropyridine site) (antagonist radioligand)
Ca2+ channel (L, diltiazem site) (benzothiazepines) (antagonist radioligand)
carbonic anhydrase II (h)
CB1 (h) (agonist radioligand)
CB2 (h) (agonist radioligand)
CCK1 (CCKA) (h) (agonist radioligand)
COX1(h)
COX2(h)
D1 (h) (antagonist radioligand)
D2S (h) (agonist radioligand)
dopamine transporter (h) (antagonist radioligand)
ERbeta Human Estrogen NHR Binding (Agonist Radioligand) Assay, Panlabs
Estrogen ER alpha (h) (agonist radioligand)
ETA (h) (agonist radioligand)
ETB (h) (agonist radioligand)
GR (h) (agonist radioligand)
H1 (h) (antagonist radioligand)
H2 (h) (antagonist radioligand)
HMG-CoA Reductase Human Enzymatic Assay, Panlabs
KATP channel (antagonist radioligand)
KV channel (antagonist radioligand)
M1 (h) (antagonist radioligand)
M2 (h) (antagonist radioligand)
MAO-A (antagonist radioligand)
MT2 (ML1B) (h) (agonist radioligand)
mu (MOP) (h) (agonist radioligand)
N neuronal alpha 4beta 2 (h) (agonist radioligand)
Na+ channel (site 2) (antagonist radioligand)
NMDA (antagonist radioligand)
norepinephrine transporter (h) (antagonist radioligand)
NTS1 (NT1) (h) (agonist radioligand)
PDE3A (h)
PDE4D2 (h)
Potassium Channel hERG (human)- [3H] Dofetilide

PPARalpha (h) (agonist radioligand)
PPARgamma h) (agonist radioligand)
PR (h) (agonist radioligand)
PXR (h) (agonist radioligand)
RARalpha (h) (agonist radioligand)
SKCa channel (antagonist radioligand)
Steroid 5 alpha-Reductase Rat Enzymatic Assay, Panlabs
TR β Human Thyroid Hormone NHR Binding (Agonist Radioligand) Assay, Panlabs
V1a (h) (agonist radioligand)

S1.8. Transcriptomics

Incubations

HepG2 (human hepatoblastoma), MCF-7 (human Caucasian breast adenocarcinoma) cells supplied by Public Health England European Collection of Cell Cultures (ECACC, Salisbury, UK). Differentiated HepaRG cells (HPR116, Lot HPR116239-TA08) were from Biopredic International (Rennes, France). Test chemicals were prepared in DMSO as serial dilutions, resulting in concentrations in media of 0.0064, 0.032, 0.16, 0.8, 4, 20 and 100 µM with 0.1% DMSO used as a vehicle control. Three biological replicates were treated with the appropriate dose of compound for 24 h prior to cell lysis. TempO-Seq analysis was performed as described previously (Yeakley et al. 2017) using the Biospyder human whole genome panel version 1 (https://biospyder.com).

Gene normalization and analysis

The raw data were processed by Sciome, USA, as described by Ebmeyer et al. (2024). Briefly, expression values for each probe were normalized by applying DESEQ2 normalization. A pseudo-read-count of 1.0 was added to each normalized expression value, and then the values were log2 transformed. Analyses performed included Gene and Pathway, Dose Response and Network Analyses.

Dose response analysis was performed using BMDExpress version 3.04. Samples with a strong monotonic increasing/decreasing trend were those which confirmed to the criteria: William's trend test adjusted p-value ≤ 0.05, Max fold change ≥ 1.5, BMDU/BMDL ≤ 40, BMD ≤ highest dose. Those genes were then used in categorical (pathway) analysis for the Gene Ontology (GO) terms and REACTOME pathways. Significant dose responsive pathways were determined according to: Fisher's exact right tail p-value ≤ 0.05, Pathway coverage ≥ 10% and number of significant dose responsive genes in pathway ≥ 3.

Supplementary Materials S2: Results for the prediction of metabolites using *in silico* models

The measured and predicted metabolites of genistein and daidzein are shown in the figures below. Possible metabolites (with priority score) of daidzein, were predicted by GLORYx (de Bruyn Kops et al., 2021) and by Meteor nexus. The green box indicates metabolites which were also identified by *in vitro* experiments. Both software programs associate the likelihood of the metabolite with a priority score.

(A) GLORYx software

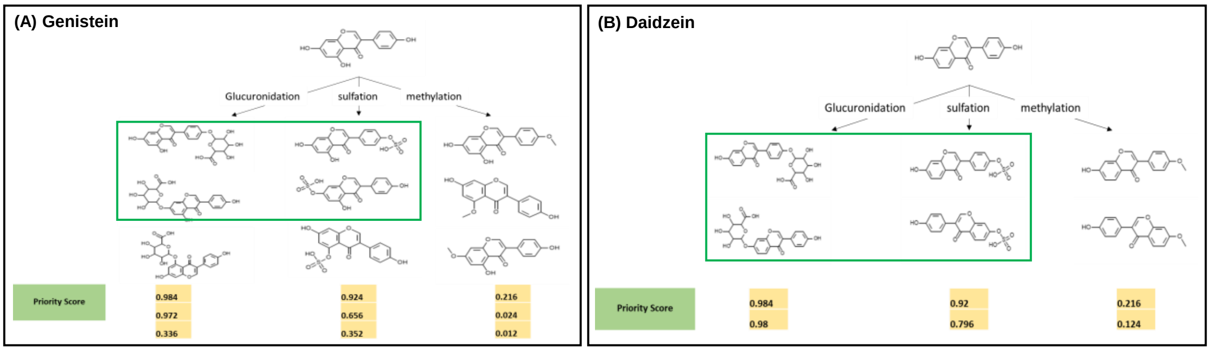

(B) Meteor nexus software

Genistein

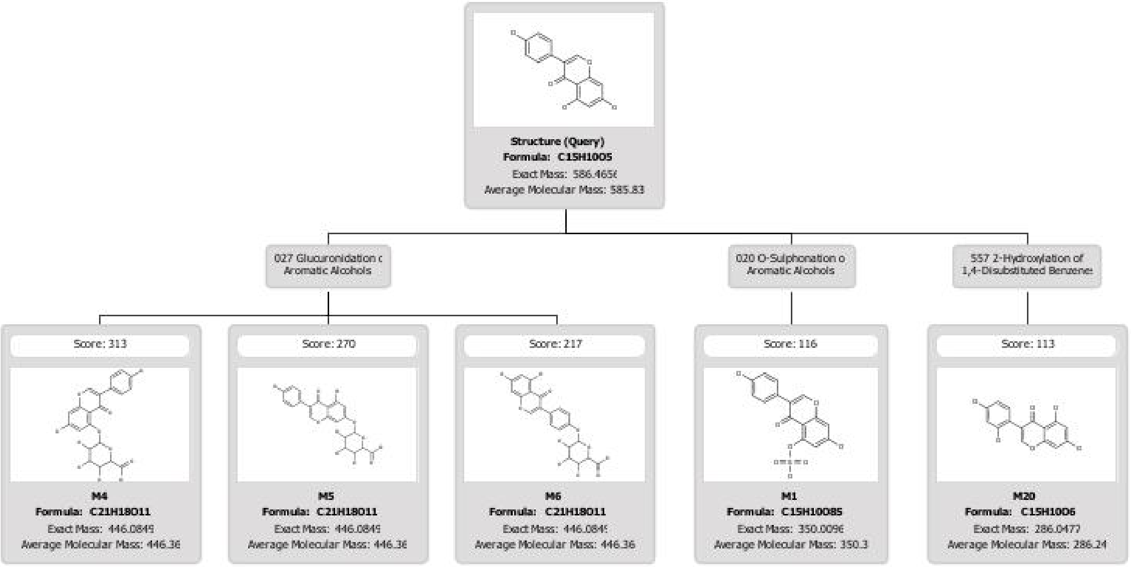

Daidzein

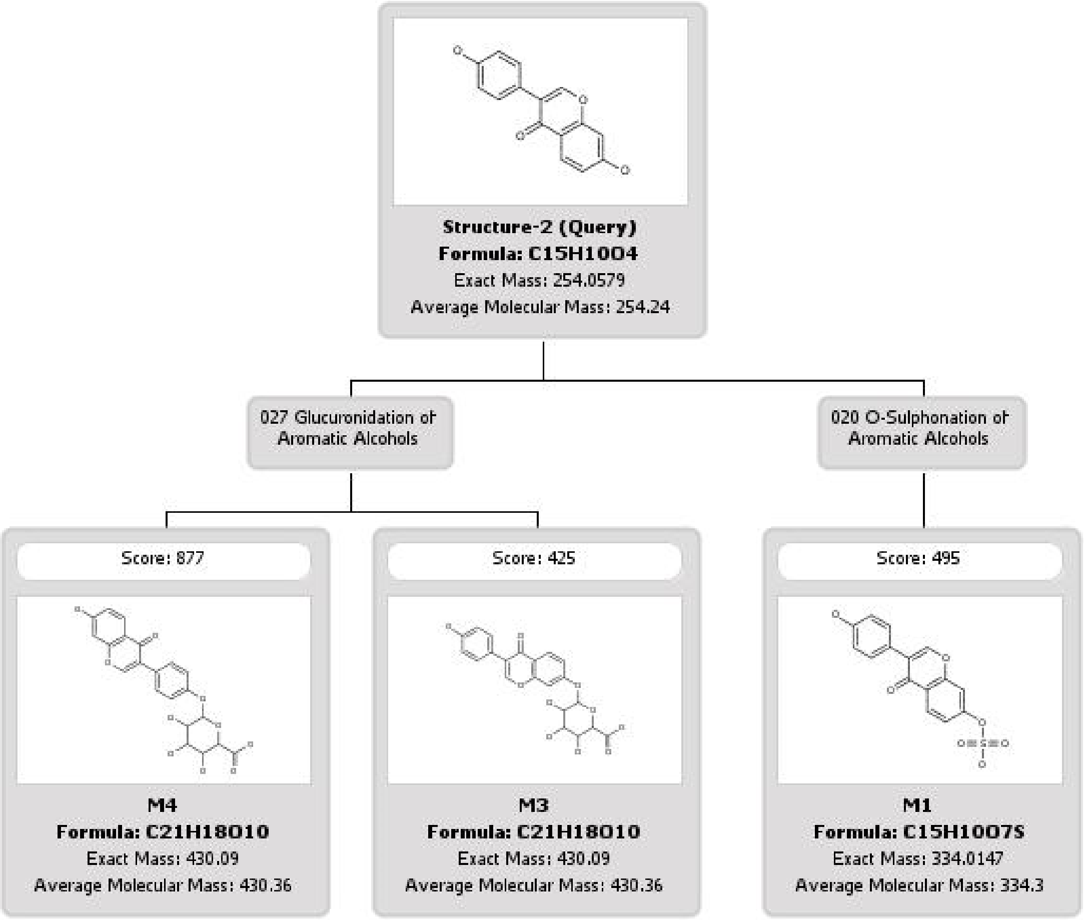
Structure-2 (Query)
Formula: C15H10O4
Exact Mass: 254.0579
Average Molecular Mass: 254.24
027 Glucuronidation of Aromatic Alcohols
020 O-Sulphonation of Aromatic Alcohols
Score: 877
M4
Formula: C21H18O10
Exact Mass: 430.09
Average Molecular Mass: 430.36
Score: 425
M3
Formula: C21H18O10
Exact Mass: 430.09
Average Molecular Mass: 430.36
Score: 495
M1
Formula: C15H10O7S
Exact Mass: 334.0147
Average Molecular Mass: 334.3

frontiers

Supplementary Materials S3: Justification of the use of genistein as a source chemical for daidzein using ToxGPS

See Excel chart

ADME CS Suppl Materials S3.xlsx

Supplementary Materials S4: Results of mutagenicity and genotoxicity assays conducted for genistein and daidzein. Information was taken from the SCCS Opinion (SCCS 2022). Note that in vivo data for daidzein were excluded for the purposes of the case study.

Mutagenicity/genotoxicity assay	**Genistein**	**Daidzein**
Ames assay	Negative in *S. typhimurium* with TA1535, TA1537, TA98 TA100 and Escherichia coli WP2 uvrA.	Inconclusive or negative results in a few Ames test studies on *S. typhimurium* TA98, TA100 and TA1538 strains.
In vitro mammalian cell chromosomal aberrations (CA)/ micronucleus (MN) tests	Of the studies of high relevance, six were positive, and one was negative.	Several studies resulted in inconclusive or negative results; however, the studies were of limited or low relevance.
In vitro mammalian cell gene mutation studies	All studies (high or limited relevance) were positive.	Inconclusive result in a mammalian cell gene mutation test (Hprt locus on V79 cells) of low relevance. Positive in limited of relevance: Na+/K+ ATPase locus on V79 and primary SHE cells.
In vitro DNA damage studies – Comet assay	Of the studies of high or limited relevance, five were positive, and four were negative.	Of the studies performed, daidzein was negative in the only one of high relevance.
Two cell transformation assay using Syrian Hamster Embryo (SHE) cells	Of the studies of high relevance, one was positive and the second was negative.	Positive in an assay of limited relevance.
In vivo chromosome aberration/ micronucleus	Negative in mice and rats after oral administration.	Not applicable (information excluded from case study)
In vivo DNA damage/Comet assay	Negative in mice after oral administration.	
In vivo gene mutation test	Several negative results in Big Blue transgenic rats: cII gene in liver, lacI gene in uterine cells, lacI gene in mammary gland cells in female, in Hprt gene in lymphocytes isolated from the spleen of female, and in lacI gene of heart tissue.	
Other *in vivo* studies	P53-/- Mouse Tumorigenesis Assay on male and female mice with genistein in the diet was negative.	

Supplementary Materials S5: *In vivo* legacy data for genistein. A subset of available *in vivo* studies relevant to reproductive toxicity and related endpoints (i.e., repeat dose toxicity) for genistein were reviewed for this case study are listed.

Reference	Study type and Description	Results	NOAEL / LOAEL (mg/kg/day)
**Repeat dose toxicity endpoint**			
Acute, subchronic and chronic safety studies with genistein in rats (McClain et al. 2006)	Genistein was administered in feed at doses of 0, 5, 50 or 500 mg/kg/day for 4 weeks in male and female rats.	No treatment-related effects on mortality, clinical signs, or ophthalmologic parameters detected. Body weight gain was reduced in males and females of the 500 mg/kg/day. Increases in adrenal weight of males and relative liver, kidney, spleen, ovary, and uterus weights of females in the 500 mg/kg/day group. Reduced seminal vesicle size was observed at necropsy in three of six males from the 500 mg/kg/day group. No treatment-related organ lesions.	Not recorded
Acute, subchronic and chronic safety studies with genistein in rats (McClain et al. 2006)	Genistein was administered in feed at doses of 0, 5, 50 or 500 mg/kg/day for 13 weeks in male and female rats.	No treatment-related deaths observed. Body weights decreased at 500 mg/kg/day group. Non-reproductive organ (heart, thyroid, kidney, and adrenal) weights weight changes in high-dose males. Testis relative weights increased in high-dose males. Relative liver and kidney weights increased in females at 500 mg/kg/day. No treatment-related gross or histopathologic alterations. With the exception of body weight effects in males, none of the treatment-related effects were observed following the 4-week recovery period.	NOAEL = 50
Acute, subchronic and chronic safety studies with genistein in rats (McClain et al. 2006)	Genistein was administered in feed at doses of 0, 5, 50 or 500 mg/kg/day for 52 weeks in male and female rats.	Several statistically significant effects on haematology and clinical chemistry parameters. Some of the hematologic effects persisted through the recovery period, but all clinical chemistry effects were resolved during recovery. Increased relative weights of adrenal and spleen (males and females), prostate, testis, ovary and uterus at 500 mg/kg/day. Increased ovary weight was the only organ weight effect that persisted through the recovery period. Watery cysts in ovaries in low-, mid-, and high-dose groups. In male rats, epididymal vacuolation was observed at 500 mg/kg/day and prostate inflammation was observed at ≥50 mg/kg/day. In female rats, histopathology alterations in ovaries and uterus/cervix at ≥50 mg/kg/day. Liver histopathology in males and females at 500 mg/kg/day.	NOAEL = 50 (based on mild hepatic effects consisting of minimal bile duct proliferation and increased γ-glutamyl transferase activity)

Subchronic and chronic safety studies with genistein in dogs (McClain et al. 2005)	Genistein was administered in feed at doses of 0, 5, 50 or 500 mg/kg/day for 4 weeks in male and female dogs.	No effect on survival, body weight gain, feed intake, ophthalmoscopy findings, clinical chemistry measurements, urinalysis endpoints, or gross or histopathologic alterations in organs.	NOAEL >500
Subchronic and chronic safety studies with genistein in dogs (McClain et al. 2005)	Genistein was administered in feed at doses of 0, 5, 50 or 500 mg/kg/day for 52 weeks in male and female dogs.	No effect on survival, body weight gain, feed intake, ophthalmoscopy findings or haematology and clinical chemistry parameters. In male dogs, testis weights were markedly decreased in 2/2 dogs of the 500 mg/kg/day group. No organ weight effects were considered statically significant or treatment-related, and none of the organ weight changes persisted through the recovery period. Transient effects of high genistein doses on the reproductive tract of dogs were functional and not considered to be adverse effects	NOAEL >500
**Reproductive toxicity endpoint**			
Multigenerational reproductive toxicology of genistein in Sprague-Dawley rats (feed study). (NTP 2008)	Genistein was administered in feed at doses of 0, 5, 100 or 500 ppm from 6 weeks until 3rd generation in male and female rats.	Female rats given 500 ppm had lower body weights, accelerated sexual maturation, and altered estrous cyclicity. Some reduction in litter size in the first two generations. Male rats given 100 or 500 ppm had increased rates of mammary gland hyperplasia and calcification of renal tubules. In the later generations, the only observed effects on offspring of exposed animals were smaller body weight gains in pups before weaning. Except for lower body weights in pups, there was no evidence for a carryover of genistein effects into unexposed generations Although genistein did show adverse effects with dietary exposures of 100 or 500 ppm, there were no clear adverse effects on the reproductive or developmental parameters measured at genistein concentrations ranging from less than 1 ppm (control diet) to 100 ppm.	**NOAEL = 5 ppm (0.3 mg/kg [males] and 0.5 mg/kg [females]**
Reproductive dose finding toxicity of genistein administered in feed to Sprague-Dawley rats (NTP 2007)	Genistein was administered to rats in feed at doses of 0, 5, 25, 100, 250, 625 or 1250 ppm from GD7 throughout	Body weight and feed consumption of treated dams prior to parturition showed decreased with increasing dose, and both parameters. Pups in the 1250 ppm group had significantly decreased body weights. Decreased ventral prostate weight in males at 1250 ppm and a trend toward higher pituitary gland to body weight ratios in both sexes. Ductal/alveolar hyperplasia of the mammary glands at >250 ppm. Ductal/alveolar hyperplasia and hypertrophy also occurred in males, with significant effects ≥25 ppm for hypertrophy and ≥250 ppm for hyperplasia. Abnormal cellular maturation (mucocyte metaplasia) in the vagina at 625 and 1250 ppm, and abnormal ovarian antral follicles at	NOAEL = 5 ppm (1.7 mg/kg/day)

	pregnancy and lactation.	1250 ppm. In males, aberrant or delayed spermatogenesis in the seminiferous tubules relative to controls at 1250 ppm. Control females showed a high incidence of renal tubule mineralization, and the severity of this lesion was significantly increased at exposure concentrations of 250 ppm or greater. Males showed no renal tubule mineralization below 250 ppm, but incidence and severity increased with increasing exposure concentration at 250 ppm and greater. No effects were observed at 5 ppm.

Supplementary Materials S6: Results from Opera predictions

The results of the prediction of the interaction of genistein and daidzein with the ER and AR are summarized below.

(A) Genistein

**Receptor**	**Experimental**	**Prediction (1=active, 0=inactive)**	**Applicability Domain (AD): (1=in, 0=out)**	**Applicability Domain Index**	**Confidence Index**
ER agonist	Active (medium)	1	1	1	1
ER antagonist	Active (weak)	1	1	1	1
ER binding	Active (medium)	1	1	1	1
AR agonist	NA	0	1	1	1
AR antagonist	NA	1	1	1	857
AR binding	NA	1	1	1	0.857

(B) Daidzein

Receptor	Experimental	Prediction (1=active, 0=inactive)	Applicability Domain (AD): (1=in, 0=out)	Applicability Domain Index	Confidence Index
ER agonist	Active (medium)	1	1	1	1
ER antagonist	NA	1	1	1	1
ER binding	Active (weak)	1	1	1	1
AR agonist	NA	0	1	1	1
AR antagonist	NA	1	1	1	816
AR binding	NA	1	1	0.307	0.547

frontiers

Supplementary Materials S7: Results from molecular docking program

Docking of (A) genistein and (B) daidzein metabolites to the estrogen, androgen, thyroid and other nuclear receptors using the open-source endocrine Disruptome tool (http://endocrinedisruptome.ki.si/). A more negative score means greater possibility for binding. GR = Glucocorticoid Receptor; LXR α = Liver X Receptor alpha; LXR β = Liver X Receptor beta; MR = Mineralocorticoid Receptor; PPAR α = Peroxisome proliferator-activated Receptor alpha; PPAR β = Peroxisome proliferator-activated receptor beta; PPAR γ = Peroxisome proliferator-activated receptor gamma; PR = Progesterone Receptor; RXR α = Retinoid X Receptor alpha; TR α = Thyroid hormone Receptor alpha; TR β = Thyroid hormone Receptor beta.

(A) Genistein

				Endocrine Disruptors																	
**Molecule Name**	**Rank**	**Priority_ score**	**Reaction**	(AR) Agonist	(AR) antagonist	(ER α) Agonist	(ER α) antagonist	(ER Receptor β)	(ER β) antagonist	GR Agonist	GR antagonist	LXR α	LXR β	MR	PPAR α	PPAR β	PPAR γ	PR	RXR α	TR α	TR β
Genistein				-9.4	-9.4	-9.4	-9.3	-8.7	-8.8	-9	-7.7	-8.9	-9.6	-9.4	-8.3	-8.6	-7.9	-2.4	-8.8	-9.7	-9.6
Molecule 1_metabolite 1	1	0.984	O-glucuronidation (aromatic_hydroxyl)	1.8	2.1	-6.6	-8.7	-0.3	-7.3	-10	-8.9	-10.2	-11	-0.1	-8.4	-8.9	-8.6	-2.9	-5.9	-6.3	-7.2
Molecule 1_metabolite 2	2	0.972	O-glucuronidation (aromatic_hydroxyl)	3.6	0.3	-6.1	-7	-0.4	-7.3	-11.2	-9	-10.6	-11	-1.3	-8.5	-8.7	-8.3	-2.7	-6.5	-8.3	-10
Molecule 1_metabolite 3	3	0.924	sulfation (aromatic_hydroxyl)	-4.6	-2.7	-6.3	-7.5	-1.2	-6.8	-9.8	-7.3	-9.4	-10	-6.2	-8.1	-8.7	-7.9	-2.1	-7.7	-8.8	-9.3

(B) Daidzein

				Endocrine Disruptors																	
**Molecule Name**	**Rank**	**Priority_ score**	**Reaction**	(AR) Agonist	(AR) antagonist	(ER α) Agonist	(ER α) antagonist	(ER Receptor β)	(ER β) antagonist	GR Agonist	GR antagonist	LXR α	LXR β	MR	PPAR α	PPAR β	PPAR γ	PR	RXR α	TR α	TR β
Daidzein				-9.3	-9.1	-9.5	-9.8	-8.7	-8.7	-9.1	-7.3	-9.1	-9.8	-9.2	-7.8	-7.9	-8.7	-2.4	-9.5	-9.5	-9.3
Molecule 1_metabolite 1	1	0.984	O-glucuronidation (aromatic_hydroxyl)	2.7	2.1	-7.1	-8.8	-0.2	-7.2	-9.8	-8.6	-10.2	-10.8	0.7	-8.1	-8.3	-8.6	-3	-6.4	-7.7	-8.8
Molecule 1_metabolite 2	2	0.98	O-glucuronidation (aromatic_hydroxyl)	minus 0	1.5	-6.4	-7.6	0.2	-7.1	-10.6	-8.8	-10.1	-11.2	1.1	-8	-8.6	-8.2	-3	-6.4	-9.4	-10
Molecule 1_metabolite 3	3	0.92	sulfation (aromatic_hydroxyl)	-5	-3.1	-6.5	-7.4	-0.9	-6.9	-9.7	-7.3	-9.7	-10.1	-5.8	-7.7	-8.2	-7.7	-2.2	-7.7	-9.3	-9.6

Probability of binding:	High Probability			low Probability

Supplementary Materials S8: Results from ToxCast

Table 2. Comparison of EATS effects by genistein and daidzein from ToxCast. Pos = positive result, Neg = negative result, Inc = uncertain/inconclusive result, NA = not applicable.

(A) Estrogen pathway

Chemical	Receptor binding		Dimerization		mRNA induction		Reporter protein Agonist		Reporter protein Antagonist		Cell Proliferation	
	Call	AC50 (µM)	Call	AC50 (µM)	Call	AC50 (µM)	Call	AC50 (µM)	Call	AC50 (µM)	Call	AC50 (µM)
Daidzein	Pos	0.17	Pos	0.77	Pos	0.15	Pos	1.68	Neg		Pos	0.15
Genistein	Pos	0.01	Pos	0.1	Pos	0.09	Pos	1.48	Neg		Pos	0.04

(B) Androgen pathway

Chemical	Receptor binding		Dimerization		mRNA induction		Reporter protein Agonist		Reporter protein Antagonist		Cell Proliferation	
	Call	AC50 (µM)	Call	AC50 (µM)	Call	AC50 (µM)	Call	AC50 (µM)	Call	AC50 (µM)	Call	AC50 (µM)
Daidzein	Neg	NA	Neg	NA	Neg	NA	Neg	NA	Inc	36.13	Neg	NA
Genistein	Neg	NA	Neg	NA	Neg	NA	Neg	NA	Neg	NA	Neg	NA

(C) Thyroid pathway

Chemical	Receptor binding (THRα)		mRNA induction		Regulation of transcription factor activity (TSHR)		Reporter protein Agonist		Reporter protein Antagonist		Thyroid peroxidase		Deiodinase (Not AOP)		Monooxygenase (Not AOP)	
	Call	AC50 (µM)	Call	AC50 (µM)	Call	AC50 (µM)	Call	AC50 (µM)	Call	AC50 (µM)	Call	AC50 (µM)	Call	AC50 (µM)	Call	AC50 (µM)
Daidzein	Neg	NA	Neg	NA	Neg	NA	Neg	NA	Neg	NA	Pos	10.25	Pos	17.25	Pos	9.1
Genistein	NA	NA	Neg	NA	Neg	NA	Neg	NA	Inc	13.14	Pos	4.49	Pos	2.43	Pos	9.44

(D) Steroidogenesis

Chemical	Aromatase	
	Call	AC50 (µM)
Daidzein	Neg	
Genistein	Neg	

Supplementary Materials S9: Results from cell stress assays

End point	Positive control and result	Genistein	Daidzein	Cell Health Parameters
AhR Translocation	Benzo(a)pyrene<br>Effect = increase<br>MEC = 0.389 µM<br>AC_{50} = >100 µM	Effect = increase<br>MEC = 51.2 µM<br>AC_{50} = > 100 µM	Effect = NR	An increase in AhR translocation into the nucleus
Nuclear size	Leflunamide<br>Effect = increase<br>MEC = 1.18 µM<br>AC_{50} = 9.87 µM	Effect = increase<br>MEC = 25.2 µM (NS)<br>AC_{50} >100 µM (NS†)	Effect = decrease<br>MEC = 69.2 µM (NS)<br>AC_{50} >100 µM (NS†)	An increase in nuclear area can indicate necrosis or G2 cell cycle arrest and a decrease can indicate apoptosis. An increase in DNA structure can indicate chromosomal instability and DNA fragmentation.
DNA structure	NA	Effect = NR	Effect = NR	An increase in DNA structure can indicate chromosomal instability and DNA fragmentation.
Cellular ATP	Rotenone<br>Effect = decrease<br>MEC <0.04 µM<br>AC_{50} = 3.05 µM	Effect = decrease<br>MEC = 31.5 µM<br>AC_{50} > 100 µM	Effect = decrease<br>MEC = 89.4 µM<br>AC_{50} > 100 µM†	Healthy cells are metabolically active generating cellular ATP, therefore a decrease in metabolically active cells will result in a decrease in the level of cellular ATP.
LDH release	Rotenone<br>Effect = increase<br>MEC = 0.138 µM<br>AC_{50} = 57.6 µM	Effect = NR	Effect = NR	An increase in LDH is due to the release of lactate dehydrogenase (LDH) from cells which have damaged membranes.
GSH content	L-BSO<br>Effect = decrease<br>MEC = 0.725 µM<br>AC_{50} = 37.5 µM	Effect = NR	Effect = NR	A decrease in glutathione (GSH) content can result from production of reactive oxygen species or from direct binding. An increase in GSH content represents an adaptive cellular response to oxidative stress.
Mitochondrial membrane potential	Rotenone<br>Effect = decrease<br>MEC = 0.00254 µM<br>AC_{50} = 0.094 µM	Effect = decrease<br>MEC = 11.6 µM<br>AC_{50} = 69.6 µM	Effect = decrease<br>MEC = 10.8 µM<br>AC_{50} = 55.3 µM	A decrease indicates a loss of mitochondrial membrane potential and mitochondrial toxicity, as well as a potential role in apoptosis signalling, an increase in mitochondrial membrane potential indicates an adaptive response to cellular energy demands.
Mitochondrial mass	Antimycin A<br>Effect = increase	Effect = increase<br>MEC = 15.0 µM	Effect = increase	A decrease in mitochondrial mass indicates loss of total mitochondria and an increase implies mitochondrial

	MEC = 00.000576 µM<br>AC_{50} = 0.245 µM	AC_{50} > 100 µM (†)	MEC = 25.9 µM (NS)<br>AC_{50} > 100 µM (NS †)	swelling or an adaptive response to cellular energy demands.
Oxidative stress (ROS)	Rotenone<br>Effect = increase<br>MEC = 0.681 µM<br>AC_{50} > 100 µM	Effect = NR	Effect = NR	An increase in reactive oxygen species (ROS) indicates the formation of toxic superoxide intermediates, an early cytotoxic response.
DNA damage: p-H2AX	(S)-(+)-camptothecin<br>Effect = increase<br>MEC = 1.06 µM<br>AC_{50} = 9.10 µM	Effect = increase<br>MEC = 13.4 µM<br>AC_{50} > 100 µM†	Effect = NR	An increase in DNA damage (p-H2AX) indicates a rise in the number of double strand breaks (DSBs). DSBs cause the phosphorylation of the histone H2AX at Ser139. DSBs are an indication of genotoxicity and can lead to apoptosis (programmed cell death).
DNA damage: Phospho-p53	Effect = increase<br>MEC = 0.412 µM<br>AC_{50} = 1.25 µM	Effect = increase<br>MEC = 22.8 µM<br>AC_{50} > 100 µM†	Effect = NR	An increase in phospho-p53 is attributed to a DNA damage response as well as other cell responses including cell cycle arrest.
ER stress panel 1: ER integrity	Tunicamycin<br>Effect = increase<br>MEC = 0.453 µM<br>AC_{50} = 2.22 µM	Effect = increase<br>MEC = 23.3 µM<br>AC_{50} > 100 µM	Effect = increase<br>MEC = 82.6 µM (NS)<br>AC_{50} >100 (NS†)	An increase in Endoplasmic Reticulum indicates endoplasmic reticulum stress
ER stress panel 1: BiP	Tunicamycin<br>Effect = increase<br>MEC < 0.391 µM (NS)<br>AC_{50} > 1.56 µM (NS)	Effect = NR	Effect = NR	An increase in BiP indicates endoplasmic reticulum stress
ER stress panel 1: XBP1	Tunicamycin<br>Effect = increase<br>MEC = 0.532 µM<br>AC_{50} > 3.13 µM	Effect = increase<br>MEC = 40.9 µM<br>AC_{50} =40.4 µM	Effect = NR	An increase in XBP1 indicates endoplasmic reticulum stress
ER stress panel 2: ATF4	Tunicamycin<br>Effect = increase<br>MEC = <0.391 µM<br>AC_{50} = 27.9 µM	Effect = NR	Effect = NR	An increase in ATF4 indicates endoplasmic reticulum stress
ER stress panel 2: PERK	Sertraline<br>Effect = increase	Effect = NR	Effect = NR	An increase in PERK indicates endoplasmic reticulum stress

	MEC = 2.17 µM (NS)<br>AC_{50} > 6.25 µM (NS)			
ER stress panel 2: CHOP	Tunicamycin<br>Effect = increase<br>MEC = 0.955 µM (NS)<br>AC_{50} > 6.25 µM (NS)	Effect = increase<br>MEC = 69.0 µM (NS)<br>AC_{50} > 100 µM (NS†)	Effect = NR	An increase in CHOP indicates endoplasmic reticulum stress
Inflammation & pH: ICAM-1	Deferoxamine mesylate salt<br>Effect = increase<br>MEC = 17.4 µM<br>AC_{50} > 200 µM (†)	Effect = increase<br>MEC = 34.4 µM<br>AC_{50} > 100 µM (†)	Effect = NR	An increase or decrease in ICAM1 indicates inflammation.
Inflammation & pH: intracellular pH	Deferoxamine mesylate salt<br>Effect = increase<br>MEC = 6.46 µM<br>AC_{50} > 200 µM (NS†)	Effect = increase<br>MEC = 50.6 µM (NS)<br>AC_{50} > 100 µM (NS)	Effect = increase<br>MEC = 26.3 µM<br>AC_{50} > 100 µM (†)	Changes in intracellular pH can indicate the interference of the compound with either the regulation of intracellular pH or the protonation of the compound itself. Specific intracellular pH is required for optimum cellular processes, distribution or target binding of the compound.
Inflammation & pH: HIF1α	Deferoxamine mesylate salt<br>Effect = increase<br>MEC = 6.2 µM<br>AC_{50} = 4.31 µM (†)	Effect = increase<br>MEC = 44.8 µM (NS)<br>AC_{50} > 100 µM (NS†)	Effect = increase<br>MEC = 32.3 µM (NS)<br>AC_{50} > 100 µM (NS)	An increase in HIF1alpha indicates hypoxia
Inflammation & pH: IL-8	Ionomycin<br>Effect = increase<br>MEC = 0.496 µM<br>AC_{50} > 10 µM (†)	Effect = NR	Effect = NR	IL-8 is a chemokine involved in inflammation and stimulation of the innate immune system. An increase in IL-8 secretion may suggest an inflammatory response.
Mitochondrial oxidative stress: PGC1α	Rosiglitazone<br>MEC = 67.8 µM (NS)<br>AC_{50} >125 µM (NS)	Effect = NR	Effect = NR	An increase in PGC1alpha indicates mitochondrial oxidative stress.
Oxidative stress: NRF2	Ethacrynic acid<br>Effect = increase<br>MEC = 3.52 µM<br>AC_{50} = 28.0 µM	Effect = increase<br>MEC = 29.9 µM<br>AC_{50} > 100 µM (NS†)	Effect = increase<br>MEC = 30.4 µM (NS)<br>AC_{50} > 100 µM (NS†)	An increase in NRF2 indicates oxidative stress.

Oxidative stress: HMOX1	Ethacrynic acid<br>Effect = increase<br>MEC = 0.824 µM<br>AC_{50} = 19.2 µM	Effect = increase<br>MEC = 29.9 (NS)<br>AC_{50} >100 (NS†)	Effect = increase<br>MEC = 44 µM<br>AC_{50} = 77.6 µM	An increase in heme oxygenase 1 (HMOX1) indicates oxidative stress.
Oxidative stress: SRXN1	Ethacrynic acid<br>Effect = increase<br>MEC = 6.17 µM<br>AC_{50} = 19.0 µM	Effect = NR	Effect = NR	An increase in SRXN1 indicates oxidative stress
Osmotic & heat shock: NFAT	Sodium chloride<br>Effect = increase<br>MEC = 1430 µM<br>AC_{50} > 80000 µM (NS†)	Effect = increase<br>MEC = 49.4 µM<br>AC_{50} > 100 (†)	Effect = increase<br>MEC = 25.0 µM<br>AC_{50} > 100 (†)	An increase in Osmotic Stress (NFAT) indicates osmotic stress.
Osmotic & heat shock: hsp70	Sorbitol<br>Effect = increase<br>MEC = 29000 µM<br>AC_{50} = 91700 µM	Effect = NR	Effect = increase<br>MEC = 3.3 µM (NS)<br>AC_{50} > 25 (NS†)	An increase in Heat Shock Response (Hsp70) indicates heat shock stress.
Metal stress: metallothionein	Cadmium (II) chloride<br>Effect = increase<br>MEC = 0.0751 µM<br>AC_{50} = 7.05 µM	Effect = NR	Effect = NR	An increase in Metallothionein indicates metal stress
Metal stress: MTF1	Cadmium (II) chloride<br>Effect = increase<br>MEC = 0.0751 µM<br>AC_{50} = 7.05 µM	Effect = decrease<br>MEC = 25.5<br>AC_{50} > 100 µM (†)	Effect = NR	An increase in MTF1 indicates metal stress
Apoptosis & necrosis: caspase 3/7	Ionomycin<br>Effect = increase<br>MEC = 0.0225 µM<br>AC_{50} = 0.798 µM	Effect = increase<br>MEC = 49.8 µM<br>AC_{50} > 100 µM (†)	Effect = increase<br>MEC = 36.9 µM (NS)<br>AC_{50} >100 (NS†)	An increase in Caspase 3/7 activity indicates the onset of the cell signalling cascade leading to cell signalled cell death (apoptosis).
Apoptosis & necrosis: cell membrane permeability	Ionomycin<br>Effect = increase<br>MEC = 1.67 µM<br>AC_{50} = 30.8 µM	Effect = increase<br>MEC = 23.8 µM (NS)<br>AC_{50} >100 (NS†)	Effect = decrease<br>MEC = 51.5 µM<br>AC_{50} = >100 †	An increase in cell membrane permeability is a general indicator of cell death.
Apoptosis & necrosis: cell cycle arrest	Ionomycin<br>Effect = decrease	Effect = decrease	Effect = NR	Determined as the ratio of G0/G1(2N) to G2/M(4N) an increase is linked to G0/G1 arrest and a decreases is

	MEC = 0.0283 µM<br>AC_{50} = 0.056 µM	MEC = 35.8 µM (NS)<br>AC_{50} >100 (NS†)		linked to G2/M arrest.
Phospholipidosis	Sertraline<br>Effect = increase<br>MEC = 0.0965 µM<br>Fold increase = 4.16	Effect = NR	Effect = increase<br>MEC = 14.2 µM (NS)<br>Fold = 1.43 (6% @ 25 µM)	An increase in phospholipidosis with a ratio of the maximum response greater or equal to 1.5 indicates an accumulation of phospholipids and/or compounds within lysosomes.
Steatosis	Sertraline<br>Effect = increase<br>MEC = 8.36 µM<br>Fold increase = 2.02	Effect = NR	Effect = increase<br>MEC = 77.4 µM (NS)<br>Fold = 1.53 (22% @ 100 µM)	An increase in steatosis indicates an accumulation of triglycerides within the cytoplasm of treated cells.
Seahorse assay: oxygen consumption rate (OCR)	Rotenone<br>Effect = decrease<br>MEC = 0.000832 µM<br>AC_{50} = 0.00432	Effect = decrease<br>MEC = 41.5 µM<br>AC_{50} > 100 µM (†)	Effect = NR	This is a measurement of oxygen content in extracellular media. Changes in OCR indicate effects on mitochondrial function and can be bi-directional. A decrease is due to an inhibition of mitochondrial respiration, whilst an increase may indicate an uncoupler, in which respiration is not linked to energy production.
Seahorse assay: Extracellular acidification rate (ECAR)	Rotenone<br>Effect = increase<br>MEC = 0.00546 µM<br>AC_{50} = 0.00549	Effect = decrease<br>MEC = 27.7 µM<br>AC_{50} > 100 µM (†)	Effect = NR	This is the measurement of extracellular proton concentration (pH). An increase in signal means an increase in rate in number of hydrogen ions (thus decreasing pH value), and seen as an increase in glycolysis. Expressed as a fraction of basal control (rate prior to addition of compound).
Seahorse assay: reserve capacity	Rotenone<br>Effect = decrease<br>MEC = 0.000657µM<br>AC_{50} = 0.00248 µM	Effect = decrease<br>MEC = 34.8 µM<br>AC_{50} > 100 µM (†)	Effect = NR	This is the measured ability of cells to respond to an increase in energy demand, a reduction indicates mitochondrial dysfunction. This measurement demonstrates how close to the bioenergetic limit the cell is.

MEC = Minimum effective concentration that significantly crosses vehicle control threshold; AC_{50} = The concentration at which 50% maximum effect is observed for each cell health parameter; † = An AC_{50} was calculated but is greater than the maximum surviving concentration; NR = No response observed; NS = Fit not statistically significant.

References

Bowes J, Brown AJ, Hamon J, et al. (2012) Reducing safety-related drug attrition: the use of in vitro pharmacological profiling. Nat Rev Drug Discov 11(12):909-22 doi:10.1038/nrd3845

Ebmeyer J, Najjar A, Lange D, et al. (2024) Next Generation Risk Assessment: An Ab Initio Case Study to assess the Systemic Safety of the Cosmetic Ingredient, Benzyl Salicylate, after Dermal Exposure. *Accepted for publicatio*n. Frontiers in Pharmacology

Hewitt NJ, Troutman J, Przibilla J, et al. (2022) Use of in vitro metabolism and biokinetics assays to refine predicted in vivo and in vitro internal exposure to the cosmetic ingredient, phenoxyethanol, for use in risk assessment. Regul Toxicol Pharmacol 131:105132 doi:10.1016/j.yrtph.2022.105132

McClain MR, Wolz E, Davidovich A, Pfannkuch F, Edwards JA, Bausch J (2006) Acute, subchronic and chronic safety studies with genistein in rats. Food Chem Toxicol 44(1):56-80 doi:10.1016/j.fct.2005.05.021

McClain RM, Wolz E, Davidovich A, Pfannkuch F, Bausch J (2005) Subchronic and chronic safety studies with genistein in dogs. Food Chem Toxicol 43(10):1461-82 doi:10.1016/j.fct.2005.02.017

NTP (2007) NTP Technical Report on the toxicology and carcinogenicity of genistein in Sprague-Dawley rats (feed study). NTP TR 545 NIH publication No. 08-4430

NTP (2008) NTP report on the multigenerational reproductive toxicology of gensitein in Sprague-Dawley rats (feed study). NTP TR 539, NIH Publication No. 08-4477.

SCCS (2022) (Scientific Committee on Consumer Safety), Scientific opinion on genistein and daidzein, preliminary version of 12 January 2022, final version of 16 September 2022, SCCS/1641/22.

Tao TP, Maschmeyer I, LeCluyse EL, et al. (2023) Development of a microphysiological skin-liver-thyroid Chip3 model and its application to evaluate the effects on thyroid hormones of topically applied cosmetic ingredients under consumer-relevant conditions. Front Pharmacol 14:1076254 doi:10.3389/fphar.2023.1076254

van Vugt-Lussenburg BMA, van der Lee RB, Man HY, et al. (2018) Incorporation of metabolic enzymes to improve predictivity of reporter gene assay results for estrogenic and anti-androgenic activity. Reprod Toxicol 75:40-48 doi:10.1016/j.reprotox.2017.11.005

Yeakley JM, Shepard PJ, Goyena DE, VanSteenhouse HC, McComb JD, Seligmann BE (2017) A trichostatin A expression signature identified by TempO-Seq targeted whole transcriptome profiling. PLoS One 12(5):e0178302 doi:10.1371/journal.pone.0178302
